# High-Altitude Medicinal Plants as Promising Source of Phytochemical Antioxidants to Combat Lifestyle-Associated Oxidative Stress-Induced Disorders

**DOI:** 10.3390/ph17080975

**Published:** 2024-07-23

**Authors:** Mohammad Vikas Ashraf, Sajid Khan, Surya Misri, Kailash S. Gaira, Sandeep Rawat, Balwant Rawat, M. A. Hannan Khan, Ali Asghar Shah, Mohd Asgher, Shoeb Ahmad

**Affiliations:** 1 Department of Biotechnology, School of Biosciences and Biotechnology, Baba Ghulam Shah Badshah University, Rajouri 185234, Jammu and Kashmir, India; vikasashraf@bgsbu.ac.in; 2 Department of Botany, School of Biosciences and Biotechnology, Baba Ghulam Shah Badshah University, Rajouri 185234, Jammu and Kashmir, India; sajidkhan717@gmail.com; 3 Section of Microbiology, School of Biosciences and Biotechnology, Baba Ghulam Shah Badshah University, Rajouri 185234, Jammu and Kashmir, India; suryamisri23@gmail.com; 4 Sikkim Regional Centre, G.B. Pant National Institute of Himalayan Environment, Pangthang, Gangtok 737101, Sikkim, India; kgaira@gmail.com (K.S.G.); sandeep_rawat15@rediffmail.com (S.R.); 5 School of Agriculture, Graphic Era University, Dehradun 24800, Utarakhand, India; balwantkam@gmail.com; 6 Department of Zoology, School of Biosciences and Biotechnology, Baba Ghulam Shah Badshah University, Rajouri 185234, Jammu and Kashmir, India; drmahkhan@bgsbu.ac.in (M.A.H.K.); aashah@bgsbu.ac.in (A.A.S.)

**Keywords:** antioxidant, high-altitude medicinal plants, lifestyle-associated disorders, oxidative stress, phytochemicals, ROS

## Abstract

Oxidative stress, driven by reactive oxygen, nitrogen, and sulphur species (ROS, RNS, RSS), poses a significant threat to cellular integrity and human health. Generated during mitochondrial respiration, inflammation, UV exposure and pollution, these species damage cells and contribute to pathologies like cardiovascular issues, neurodegeneration, cancer, and metabolic syndromes. Lifestyle factors exert a substantial influence on oxidative stress levels, with mitochondria emerging as pivotal players in ROS generation and cellular equilibrium. Phytochemicals, abundant in plants, such as carotenoids, ascorbic acid, tocopherols and polyphenols, offer diverse antioxidant mechanisms. They scavenge free radicals, chelate metal ions, and modulate cellular signalling pathways to mitigate oxidative damage. Furthermore, plants thriving in high-altitude regions are adapted to extreme conditions, and synthesize secondary metabolites, like flavonoids and phenolic compounds in bulk quantities, which act to form a robust antioxidant defence against oxidative stress, including UV radiation and temperature fluctuations. These plants are promising sources for drug development, offering innovative strategies by which to manage oxidative stress-related ailments and enhance human health. Understanding and harnessing the antioxidant potential of phytochemicals from high-altitude plants represent crucial steps in combating oxidative stress-induced disorders and promoting overall wellbeing. This study offers a comprehensive summary of the production and physio-pathological aspects of lifestyle-induced oxidative stress disorders and explores the potential of phytochemicals as promising antioxidants. Additionally, it presents an appraisal of high-altitude medicinal plants as significant sources of antioxidants, highlighting their potential for drug development and the creation of innovative antioxidant therapeutic approaches.

## 1. Introduction

Oxidative stress (OS) within organisms arises when there is an imbalance between the production of reactive oxygen species (ROS) and the body’s ability to neutralize them [1]. ROS are generated during cellular metabolism, particularly in processes like the respiratory chain and tricarboxylic acid (TCA) cycle within mitochondria. ROS, including hydrogen peroxide (H_2_O_2_) and superoxide anion (O_2_^•−^), play essential roles in physiological functions such as cellular defence and signalling [2]. However, disproportion between ROS production and neutralization can lead to oxidative stress, which is implicated in various pathological conditions [3]. This imbalance, where an excess of reactive molecules overwhelms the body’s innate defence mechanisms, damages cellular structures and essential molecules like lipids, proteins and DNA. As a result, this leads to the development and progression of multiple diseases [4]. While ROS, when present in controlled, low concentrations, serve as signalling molecules facilitating cellular functions and offering cellular protection, their excessive production, as seen in conditions like inflammation, can spur the generation of additional highly reactive species, such as superoxide radical (O_2_^•−^), hydroperoxyl radical (HO_2_), singlet oxygen (1O_2_), ozone (O_3_), nitric oxide (NO), nitrogen dioxide (NO_2_), sulphur dioxide (SO_2_), and sulphur trioxide (SO_3_) [5]. These reactive species react with cellular components, modifying their normal structure and function. Notably, the oxidative modification of essential enzymes or regulatory sites is critical, changing their redox potential, that trigger alterations in cell signalling pathways and induce programmed cell death [6]. Evidently, oxidative stress and inflammation are closely linked. Oxidative stress can trigger inflammation, while inflammation can, in turn, amplify OS. This creates a harmful cycle that promotes cell damage and a pro-inflammatory environment [7].

**Figure 1:**
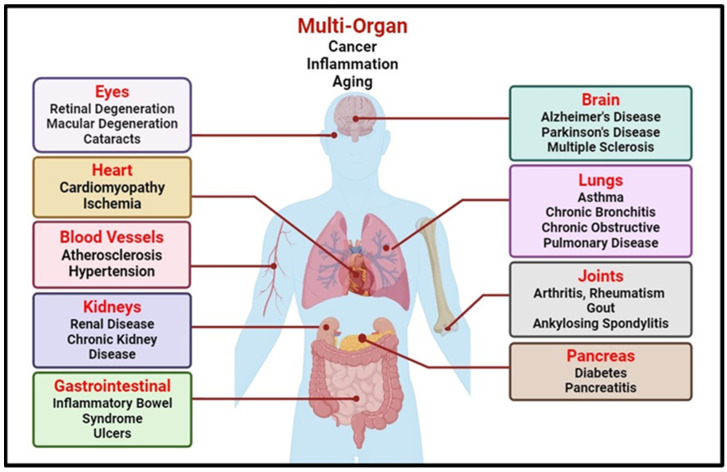
Oxidative stress-induced health modalities (The illustration was created using BioRender at www.biorender.com).

Oxidative stress stands as a central mechanism in the pathogenesis of a spectrum of health disorders, spanning cardiovascular, neurodegenerative, and metabolic conditions such as obesity, diabetes and many others [8] (Figure 1). Its pivotal role is evident in the disruption of cell membrane integrity through induced lipid peroxidation, contributing significantly to the progression of cardiovascular complications like atherosclerosis, endothelial dysfunction, and plaque formation, as well as neuronal membrane damage, which underlies various neurodegenerative diseases [9].

Moreover, the impact of ROS extends beyond membrane disruption, influencing critical proteins and enzymes and thereby compromising essential cellular functions and signalling pathways. This includes the matrix metalloproteinases (MMPs) activation in cardiovascular ailments and the initiation of protein misfolding and aggregation, characteristic of neurodegenerative disorders like Parkinson’s and Alzheimer’s diseases [10]. Furthermore, oxidative stress triggers an inflammatory cascade, marked by the release of pro-inflammatory mediators, perpetuating a cycle that exacerbates cellular damage and disease progression. Notably, oxidative stress causes adipose tissue inflammation and dysfunction, increasing pro-inflammatory cytokines and adipokines, which lead to insulin resistance and disrupted lipid metabolism leading to obesity [11]. In diabetes, oxidative stress impairs insulin signalling and damages pancreatic β-cells, reducing glucose uptake and insulin secretion, thereby worsening the disease [12]. Understanding the intricate interplay between oxidative stress and its associated inflammatory responses is paramount, as it not only elucidates the underlying mechanisms of disease but also offers promising avenues for therapeutic intervention in combating these debilitating health conditions.

Phytochemicals, particularly those derived from high-altitude medicinal plants, have emerged as potent antioxidants with the potential to counteract oxidative stress and its associated health disorders by scavenging harmful free radicals in the body [13]. Their diverse mechanisms of action also include anti-inflammatory effects, modulation of cellular signalling pathways, and enhancement of immune function. High altitude medicinal plants have adapted to extreme environmental conditions such as low oxygen levels, intense ultraviolet radiation and temperature fluctuations. These harsh conditions stimulate the production of bioactive compounds within these plants, making them rich sources of phytochemicals with unique properties [14]. The exploration of high-altitude medicinal plants not only preserves cultural traditions but also harnesses their therapeutic potential for modern medicine, particularly in combating oxidative stress-related diseases and discovering novel pharmaceutical compounds. Though high-altitude regions harbour a vast array of plant species and genetic diversity, much of this biodiversity remains unexplored and underutilized [15]. This untapped reservoir of biological diversity offers immense potential for discovering new bioactive compounds and understanding evolutionary adaptations to extreme environments. Therefore, exploring high-altitude medicinal plants as sources of potent antioxidants not only advances our understanding of natural defence mechanisms but also paves the way for developing innovative therapeutic strategies to overcome oxidative stress-related diseases [16]. This study highlights and summarizes the production and physio-pathological aspects of oxidative imbalance and emphasizes the role of phytochemicals in mitigating these effects. Further, this study provides a comprehensive tabulation of more than 160 high-altitude medicinal plants along with their reported phytochemicals, which could be very useful in harnessing their potential to combat lifestyle-associated, oxidative stress-induced disorders and could serve as a starting point for the exploration of alternate medicine for combating these diseases.

### 1.1. Oxidative Stress: Source, Mechanism and Lifestyle-Related Diseases

#### 1.1.1. Source of Oxidative Stress 

Oxidative stress occurs when highly reactive species, such as superoxide radical (O_2_^•−^), hydroperoxyl radical (HO_2_), singlet oxygen (1O_2_), and ozone (O_3_); reactive nitrogen species (RNS) like nitric oxide (NO) and nitrogen dioxide (NO_2_); and reactive sulphur species (RSS) like sulphur dioxide (SO_2_) and sulphur trioxide (SO_3_), overwhelm the natural antioxidant defence system of a body. This leads to cellular damage and dysfunction, which can contribute to a wide array of diseases [17]. These reactive species are continuously produced within cells at low levels during normal metabolic processes, which are safely neutralized by cellular machinery, but can also stem from contact to external factors such as radiation (such as X-rays and UV), air pollutants, ozone, cigarette smoke, bacteria, viruses, drugs and various forms of cellular stress, whether acute or chronic [18].

These reactive species include both non-radicals and free radical oxidants. Free radicals are particularly unstable due to having unpaired electrons in their outer electron orbit. This instability drives them to react with other molecules, causing oxidation and subsequent harm to crucial biological molecules such as nucleic acids (DNA, RNA), lipids and proteins [19].

The key intercellular origin of these reactive species includes endoplasmic reticulum, mitochondria, peroxisomes, lysosomes, plasma membrane and, cytosol [20]. ROS, formed from the chemical reactions involving molecular oxygen, encompass free radicals such as superoxide anions (O_2_^•−^) and hydroxyl radicals (OH⁻), alongside non-radical oxidants like hydrogen peroxide (H_2_O_2_) and hypochlorous acid (HOCl). Reactive nitrogen species, on the other hand, include peroxynitrite radical (ONOO⁻), and nitric oxide (NO). Recently identified reactive sulphur species (RSS) include thiol radical (RS^•^) and RSS formed through reactions between ROS and thiols. RSS exhibit both radical and non-radical properties, and they have a particular affinity for sulphur-containing molecules, such as peptides and proteins, triggering oxidation and reduction reactions [20].

Enzymes of the mitochondrial electron transport respiratory chain are major contributors to ROS production [21]. Furthermore, various other enzymes catalyse chemical reactions that contribute to ROS formation. These include homologs of phospholipase A2 (PLA2), nicotinamide adenine dinucleotide phosphate (NADPH) oxidase, cyclooxygenase (COX), uncoupled nitric oxide (NOS), xanthine oxidase (XO), glucose oxidase (GOXs), myeloperoxidase (MPO) and, lipoxygenases (LOXs) [22]. 

NADPH oxidase (NOX), initially identified in the phagosomes of immune cells, has several homologs with diverse intracellular localizations. Some homologs, like DUOX2 and NOX1, play major roles in various inflammatory conditions and tumours. Xanthine oxidase, primarily expressed in the small intestinal mucosa and liver, catalyses ROS production both on the outer surface of the plasma membrane and in the cytoplasm [23]. Lipoxygenases are non-heme iron enzymes that accumulate ROS by oxidizing arachidonic acid (AA), whereas myeloperoxidase, a heme protein that localizes lysosomes, contributes to ROS production in immune cells [24].

#### 1.1.2. Mechanism of ROS Production

**Figure 2:**
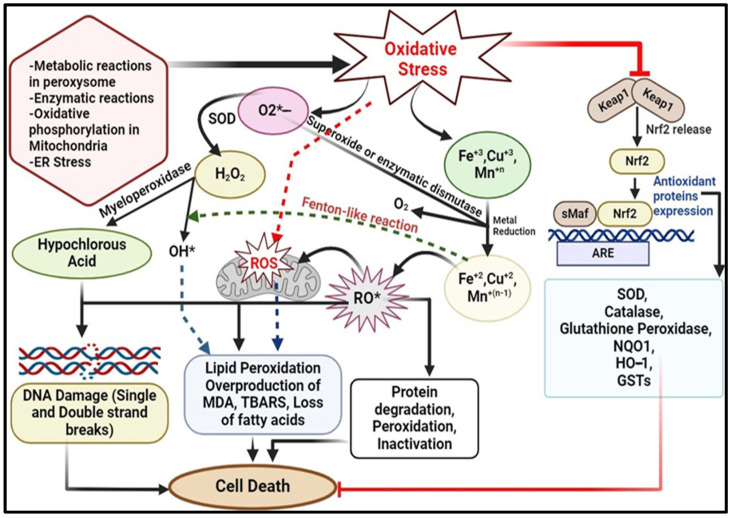
Source, mechanism of production of ROS leading to oxidative stress and its repercussions along with cellular antioxidant defence. (The illustration was created using BioRender www.biorender.com.) [*: Free radical; Nrf2: Nuclear factor erythroid 2-related factor 2; ARE: Antioxidant Response Element; Mn: Manganese; Cu: Copper; Fe: Iron; OH: Hydroxyl radical; SOD: Superoxide Dismutase; NQO1: NAD(P)H quinone dehydrogenase 1; HO-1: Heme Oxygenase-1; GSTs: Glutathione S-transferases; MDA: Malondialdehyde; TBARS: Thiobarbituric Acid Reactive Substances; ROS: Reactive Oxygen Species; RO: Reactive Oxygen; O_2_: Oxygen; Keap1: Kelch-like ECH-associated protein 1; sMaf: Small Maf proteins; H_2_O_2_: Hydrogen Peroxide].

Mitochondria is the main endogenous source of ROS because of its involvement in ATP synthesis through oxidative phosphorylation. This process involves the reduction of molecular oxygen (O_2_) to water (H_2_O) in the electron transport chain (ETC) [10]. Superoxide (O_2_^•−^) production within mitochondria is a significant contributor to cellular ROS. Seven primary sites of superoxide production have been identified in mammalian cells [6]. Ranked by their highest capacity, these include the ubiquinone binding sites in complex I (site IQ) and complex III (site IIIQo); glycerol 3-phosphate dehydrogenase (GPDH); the flavin in complex I (site IF); electron transferring flavoprotein: Q oxidoreductase (ETFQOR), involved in fatty acid beta-oxidation; and pyruvate and 2-oxoglutarate dehydrogenases. Most of these complexes release O_2_^•−^ into the mitochondrial matrix, except for complex III site and GPDH. Within the mitochondrial membrane, three types of superoxide dismutase (SOD) exist: copper superoxide dismutase (Cu-SOD), manganese superoxide dismutase (Mn-SOD), and zinc superoxide dismutase (Zn-SOD). Mn-SOD catalyses the conversion of O_2_^•−^ into H_2_O_2_. Hydrogen peroxide can then be converted into a hydroxyl radical by the enzyme aconitase through the Fenton reaction. Copper and zinc SODs function primarily in the inter-membrane space to convert superoxide into less ROS [25] (Figure 2).

An alternative pathway for generating ROS involves the mitochondrial cytochrome catalytic cycle, which includes enzymes like cytochrome P450. These enzymes process a broad variety of organic compounds, such as steroids, lipids and, xenobiotics, leading to the production of different reactive byproducts, including hydrogen peroxide and superoxide radicals [2]. Additionally, in mammals, various protein complexes, such as nicotinamide adenine dinucleotide (NADH)-cytochrome b5 reductase (b5R), dihydroorotate dehydrogenase (DHODH), succinate dehydrogenase (SDH) from complex II, and monoamine oxidases (MAO), generate ROS [5]. Numerous antioxidant defence systems safeguard mitochondria from the detrimental effects of ROS. These include endogenous antioxidants like glutathione peroxidases (GPXs), thioredoxin peroxidases (TRXPs), SODs, peroxiredoxins (PRDXs), glutathione (GSH), thioredoxin-2 (TRX2), glutaredoxin-2 (GRX2), cytochrome C oxidase (complex IV), and coenzyme Q. Additionally, exogenous antioxidants, such as ascorbic acid, vitamin E, and phytochemicals (carotenes, phenols, etc.), play crucial roles in this protective mechanism [5] (Figure 2). Excessive production of ROS is associated with numerous human disorders. These include myocardial dysfunction, inflammation, diabetes, neurodegenerative disease, aging, chronic kidney disease and DNA damage leading to cancer. ROS can cause damage to genomic and mitochondrial DNA, leading to mutations in somatic cells, genomic instability, activation of oncogenes, suppression of tumour suppressor genes, and disruptions in various metabolic and signalling pathways. Compensatory mechanisms may initially be activated but can ultimately contribute to cellular damage and the development of various pathological conditions [2].

#### 1.1.3. Lifestyle-Associated Oxidative Stress-Induced Disorders

Besides many other external factors, lifestyle factors, such as lack of physical activity, smoking, poor dietary habits, and excessive alcohol intake, predominately sponsors the onset of oxidative stress-related disorders (Figure 1). These behaviours result in the overproduction of ROS, overwhelming the body’s antioxidant defences and leading to oxidative stress [26]. In cardiovascular diseases, oxidative stress damages blood vessels and promotes atherosclerosis. In neurodegenerative disorders, ROS-induced neuronal damage accelerates conditions like Parkinson’s and Alzheimer’s diseases [27]. For metabolic disorders, oxidative stress disrupts insulin signalling and lipid metabolism, fostering obesity and diabetes [28]. Addressing these lifestyle factors is crucial for preventing and managing these oxidative stress-related diseases.

##### Cardiovascular Diseases

1. Atherosclerosis

Oxidative stress stages the oxidation process of LDL cholesterol, giving rise to oxidized LDL (oxLDL). Within the arterial wall, macrophages ingest oxLDL, which triggers foam cell formation and initiates an inflammatory reaction. This response triggers the release of chemokines, and cytokines, which recruit additional immune cells to the site of inflammation. Further, oxidative stress enhances endothelial dysfunction, promoting vasoconstriction and platelet aggregation, which contribute to plaque formation and narrowing of arteries [29].

2. Hypertension

Oxidative stress diminishes the availability of nitric oxide (NO), a powerful vasodilator, by scavenging it and promoting its inactivation. This results in endothelial dysfunction and impaired vasodilation, contributing to increased peripheral vascular resistance and hypertension. Moreover, ROS can activate the renin—angiotensin—aldosterone system (RAAS), that leads to vasoconstriction and sodium retention, further exacerbating hypertension [30].

3. Myocardial Infarction

Oxidative stress stages the development and progression of plaque and atherosclerosis instability, thereby increasing the risk of plaque rupture and thrombosis. ROS can directly damage cardiomyocytes and impair myocardial contractility. Additionally, oxidative stress activates inflammatory pathways, promoting myocardial inflammation and fibrosis, which can lead to cardiac remodelling and dysfunction [31].

##### Neurodegenerative Diseases

1. Alzheimer’s Disease (AD)

Oxidative stress induces the accumulation of hyperphosphorylated *tau* proteins and, β-amyloid (Aβ) peptides, leading to the formation of senile plaques and neurofibrillary tangles, respectively. ROS also disrupts calcium homeostasis, mitochondrial function, and synaptic transmission, contributing to neuronal dysfunction and cell death. Inflammatory mediators, including cytokines and microglial activation, further exacerbate neuroinflammation and neuronal damage in AD [32].

2. Parkinson’s Disease (PD)

Oxidative stress promotes the misfolding and accumulation of α-synuclein protein, forming Lewy bodies, the pathological hallmark of PD. ROS-induced mitochondrial dysfunction leads to impaired energy production, increased oxidative damage, and neuronal cell death, particularly in dopaminergic neurons of the substantia nigra. Additionally, oxidative stress activates microglia and astrocytes, triggering neuroinflammation and neurodegeneration in PD [33].

##### Cancer

1. DNA Damage and Mutation

Oxidative stress induces DNA lesions, including strand breaks, base modifications and DNA—protein cross-links. Unrepaired DNA damage can lead to mutations in tumour suppressor genes and oncogenes, promoting the initiation and progression of tumours. Additionally, ROS-mediated activation of signalling pathways, such as nuclear factor-kappa B (NF-κB) and mitogen-activated protein kinases (MAPKs), further drives tumour growth, invasion, and metastasis [34].

2. Tumour Angiogenesis

Oxidative stress promotes the production of angiogenic factors, such as vascular endothelial growth factor (VEGF) and hypoxia-inducible factor 1-alpha (HIF-1α), which stimulate the formation of new blood vessels to support tumour growth and metastasis. ROS-mediated activation of pro-angiogenic pathways and inhibition of anti-angiogenic factors contribute to tumour angiogenesis and neovascularization [35].

##### Metabolic Disorders

1. Insulin Resistance

Oxidative stress impairs insulin signalling pathways by promoting serine phosphorylation of insulin receptor substrate 1 (IRS-1), inhibiting its association with the insulin receptor and downstream activation of phosphatidylinositol 3-kinase (PI3K) and Akt. This leads to decreased glucose uptake and glycogen synthesis, and increased gluconeogenesis and lipolysis, leading to insulin resistance and hyperglycaemia in type 2 diabetes [36].

2. Obesity

Oxidative stress promotes adipocyte dysfunction and inflammation by activating pro-inflammatory pathways, such as NF-κB and c-Jun N-terminal kinase (JNK). ROS induce the secretion of pro-inflammatory cytokines, such as tumour necrosis factor-alpha (TNF-α) and interleukin-6 (IL-6), from adipose tissue macrophages and adipocytes, causing a persistent inflammatory condition that leads to insulin resistance, dyslipidemia, and overall metabolic dysfunction [37].

### 1.2. Antioxidant Defence Systems

Antioxidants play a pivotal part in preventing or delaying the oxidation of target molecules caused by ROS, which in turn leads to oxidative stress. These compounds act as defenders by donating electrons to free radicals, neutralizing their harmful effects on lipids, proteins, DNA, and other biomolecules [38]. They serve as scavengers within biological systems and are essential defence mechanisms against oxidative stress [4].

**Figure 3:**
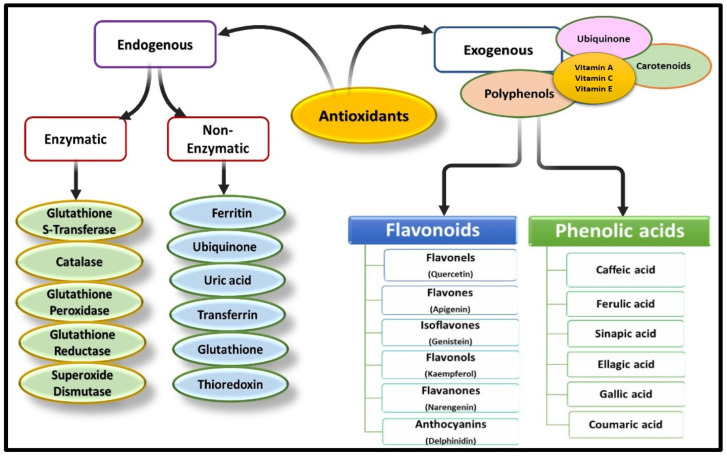
Endogenous and exogenous sources of antioxidants. (The illustration was created using BioRender www.biorender.com).

Antioxidants can originate from external sources, known as exogenous antioxidants, which are mainly obtained through food, as well as from internal sources, referred to as endogenous antioxidants, which are produced within the body [39]. Endogenous antioxidants can be enzymatic or non-enzymatic in nature [40]. Enzymatic antioxidants are a specific category of antioxidant systems present in the human body. These enzymes possess antioxidant activity and are capable of acquiring different valences, allowing them to transfer electrons to neighbouring free radicals, thereby facilitating their breakdown and neutralization [41]. Some examples of enzymatic antioxidants include glutathione reductase (GR), superoxide dismutase, catalase (CAT), and glutathione peroxidase (GPx) [42]. Glutathione reductase aids in the production of reduced glutathione, which helps counteract the oxidative damage caused by ROS [43]. Similarly, SOD plays a crucial role in neutralizing free radical species by converting superoxide radicals into hydrogen peroxide [25,44]. Non-enzymatic endogenous antioxidants are produced within the body through various metabolic pathways and physiological processes. Therefore, these antioxidants are essential for neutralizing ROS and protecting cells from oxidative damage [45]. Some examples of non-enzymatic endogenous antioxidants are glutathione (GSH), uric acid, bilirubin, melatonin and alpha-lipoic acid (Figure 3). 

Exogenous antioxidants refer to the types of antioxidants that originate outside the body and can be supplied to the body primarily through diet or supplements. These antioxidants encompass various essential nutrients like vitamin C, vitamin E, omega-3 and omega-6 fatty acids [46]. Additionally, they may include certain plant-derived phytochemicals such as polyphenols, including flavonoids, as well as trace elements like zinc and manganese [16]. Synthetic antioxidants like butyl hydroxyanisole may also be classified as exogenous antioxidants, as they aid in preventing lipid oxidation [40].

Phytochemicals are low molecular weight non-enzymatic compounds produced by plants and possess numerous medicinal and therapeutic properties [47,48]. Certain phytochemicals possess antioxidant properties and actively engage with oxidative radicals, neutralizing their harmful effects through various mechanisms. These include scavenging free radicals by electron transfer and chelating metal ions that trigger ROS production. Different groups of phytochemicals such as flavonoids, ascorbic acid and carotenoids, exhibit diverse antioxidant activities against different ROS.

Medicinal plants that thrive at high altitudes possess inherent protective processes against the detrimental results of ROS [49]. They produce enzymatic antioxidants like SOD and CAT, as well as non-enzymatic antioxidants such as tannins, flavonoids, and ascorbic acid in bulk quantities to mitigate harsh environmental stress factors [50]. However, due to challenges associated with their isolation and the risk of denaturation, plant-derived enzymatic antioxidants are typically not employed for therapeutic purposes [51]. Some plants possess genetic capabilities to synthesize phytochemicals that effectively neutralize toxic ROS [47]. Additionally, exposure to various environmental stresses stimulates the production of phytochemicals, which act as countermeasures against ROS [50]. These secondary metabolites, derived from essential metabolic pathways, exert protective effects by preventing the oxidation of plant proteins, lipids, and DNA through passive or active resistance mechanisms [52].

This study provides a summary of major oxidative stress-induced health disorders and mechanistic details of phytochemicals being used as antioxidants. This study also aims to focus upon high altitude medicinal plants as the bulk producers of antioxidants and as a potential source of plant-derived therapeutic agents against lifestyle-induced oxidative stress-related diseases.

## 2. Phytochemicals as Antioxidants

Phytochemicals are non-enzymatic compounds, with low molecular weight, that abundantly exist in plants [53]. These biologically active substances have gained recognition for their medicinal and therapeutic properties. The World Health Organization (WHO) has acknowledged the use of these plant-derived compounds in the treatment of various human diseases, highlighting their significance in healthcare [48]. Numerous phytochemicals possess antioxidant properties and actively engage with oxidative radicals such as ROS, neutralizing their harmful effects by scavenging free radicals by electron transfer and chelating metal ions that trigger ROS production [47]. Many phytochemicals, such as flavonoids, ascorbic acid and carotenoids, show diverse mechanisms by which to counter the effects of ROS and to therefore mitigate OS [13]. These phytochemicals offer immense potential for inhibiting and treating oxidative stress, contributing to the overall wellbeing and health of individuals.

### 2.1. Carotenoids

Carotenoids are lipophilic pigments found in plant plastids. They are responsible for the vibrant colours seen in various fruits and vegetables [54]. Carotenes, having a beta-ionone ring, also serve as a crucial source for the synthesis of vitamin A [55]. Almost 1200 natural carotenoids have been identified and characterized so far, along with their structures and biological sources (http://carotenoiddb.jp; accessed on 7 June 2024), with beta-carotene being the most extensively studied among them [56]. The chemical structure of carotenoids consists of 40 carbon atoms arranged in a specific pattern of double bonds, which contributes to their antioxidant properties [57].

Carotenoids can be broadly classified into two categories: carotenes, which contain carbon and hydrogen atoms, and xanthophylls, which contain at least one oxygen atom [58]. Carotenes include alpha-carotene, beta-carotene, lutein, and lycopene, while xanthophylls encompass canthaxanthin, antheraxanthin, zeaxanthin, and others [59].

The antioxidant action of carotenoids primarily involves their ability to react with peroxyl radicals and singlet oxygen species, thereby preventing oxidative damage to lipid membranes [60]. Singlet oxygen species transfer their energy to nearby carotenoid molecules, allowing the oxygen molecule to return to its non-toxic state. The excited carotenoid molecule then dissipates its energy to the surrounding solvent, returning to its ground state and enabling it to react with other free radicals [61].

Carotenoids have demonstrated effectiveness against various diseases associated with oxidative stress, including Alzheimer’s disease [62]. Certain carotenoids, such as beta-carotene, have been found to bind efficiently to receptors associated with Alzheimer’s disease, such as histone and p53 receptors [63]. Carotenoids also play a protective role against photo-oxidative damage to the skin caused by UV radiation. By leveraging their antioxidant properties, carotenoids, like lycopene and beta-carotene, can help suppress and inhibit skin diseases, mitigating the risk of dermatoses and cutaneous malignancy [60]. Additionally, carotenoids show potential in inhibiting the progression of health abnormalities such as rheumatoid arthritis and have cardiovascular protective effects [64]. Lutein and zeaxanthin, key carotenoids concentrated in the macula of the eye, play critical roles in eye health by acting as antioxidants and blue light filters. These compounds protect retinal cells by neutralizing ROS and reducing oxidative stress, which are known contributors to age-related macular degeneration (AMD). Mechanistically, lutein and zeaxanthin absorb blue light wavelengths, particularly those most damaging to the retina (400—500 nm), thereby preventing phototoxicity and subsequent cellular damage. Their presence in the macular pigment also enhances visual performance by improving contrast sensitivity and by reducing glare. Scientific evidence supports their effectiveness in maintaining retinal integrity and potentially slowing the progression of AMD, underscoring their importance in preserving long-term eye function and vision [65].

Overall, carotenoids serve as valuable antioxidants, contributing to the prevention and management of various diseases linked to oxidative stress.

### 2.2. Ascorbic Acid (AsA)

Ascorbic acid (AsA), popularly known as vitamin C, plays an important role in the non-enzymatic defence mechanisms against ROS [66]. This class of antioxidant compounds consists of low molecular weight substances that act as reducing agents [67]. Plants produce ascorbic acid through the Smirnoff-Wheeler pathway, involving the conversion of mannose and lactose in their _D_ and _L_ forms. Additionally, the Wolucka—Van pathway serves as an alternative route for synthesizing ascorbic acid in plants. Mitochondria, particularly in the photosynthetic tissues of plants, serve as key sites for the production of ascorbic acid, which exists in two forms: semi-dehydroascorbyl radical and dehydroascorbate [68].

Ascorbic acid (vitamin C) plays a pivotal role in the ascorbate—glutathione cycle in plants, serving as a primary antioxidant by scavenging ROS such as hydrogen peroxide [69]. It undergoes oxidation to monodehydroascorbate (MDHA) and dehydroascorbate (DHA) during ROS detoxification. DHA is then reduced back to ascorbic acid by dehydroascorbate reductase (DHAR), with the assistance of glutathione, thereby replenishing the cellular pool of active ascorbate. Additionally, ascorbic acid regenerates oxidized vitamin E (tocopherol and tocotrienol) by reducing tocopheroxyl radicals (vitamin E), prolonging vitamin E’s antioxidant function in protecting cellular membranes from oxidative damage. This cycle ensures effective antioxidant defence and redox homeostasis, essential for plant resilience against environmental stressors [70].

Within plants, free radicals are generated as a result of metabolic activities in the presence of oxygen or exposure to UV radiation [19]. Ascorbic acid acts as an antioxidant by scavenging ROS, including hydrogen peroxide, superoxide anion, and hydroxyl radical, forming monodehydroascorbate. By doing so, it protects essential biomolecules such as unsaturated fatty acids, proteins, and DNA from damage [71]. The antioxidant activity of ascorbic acid contributes to the prevention of various cardiovascular disorders and gastric problems. It enhances the concentration of nitric oxide in the vascular endothelium, thus aiding in the prevention of hypertension. Moreover, ascorbic acid promotes the absorption of iron in the small intestine, offering potential inhibition of gastric issues associated with *Helicobacter pylori* infection [72].

Overall, ascorbic acid serves as a vital antioxidant in plants, safeguarding against oxidative damage and contributing to the prevention of cardiovascular and gastric ailments in humans [73].

### 2.3. Tocopherols and Tocotrienols

Tocopherols and tocotrienols are isoforms of vitamin E, consisting of four types: alpha, beta, gamma, and delta [74]. These phytochemicals possess a hydrophobic nature and contain a prenyl group [75]. They exhibit significant antioxidant activity and play a crucial role in preventing various cardiovascular diseases, neurodegenerative diseases, like Alzheimer’s, and aging [76]. The antioxidant characteristics of tocopherols and tocotrienols are attributed to the occurrence of a chromanol ring in their structure. This ring contains a hydroxyl group that combats free radicals by donating hydrogen atoms [77]. 

Among the various forms of vitamin E, both alpha tocopherols and tocotrienols are particularly active in preventing lipid peroxidation caused by free radicals, thereby protecting cell membranes from damage [78]. The alpha forms of tocopherols and tocotrienols work by inhibiting the generation of free radicals, while the gamma forms are effective in capturing and neutralizing the impacts of ROS. Collectively, these vitamin E isoforms contribute to the body’s defence against oxidative stress and its detrimental effects [77].

### 2.4. Polyphenols

Polyphenols are a prominent class of phytochemicals, which play a major role as antioxidants [79]. They are synthesized by plants as a result of shikimic acid pathway from amino acids phenylalanine or tyrosine [80]. Polyphenols exhibit varying molecular weights depending upon the degree of polymerization (small molecules such as quercetin have a molecular weight of 302.24 Da, while, as tannins, they can reach several thousand kDa due to their polymeric nature) and exert antioxidant effects by acting as reducing agents [81]. They donate hydrogen atoms to the ROS produced, thus, scavenging the free radical species [82]. Important polyphenols present in the plants, which perform antioxidant activity, are flavonoids, phenolic acids and lignans [83,84,85].

Flavonoids are a major group of plant phenolic compounds, characterized by the presence of a flavan nucleus in their chemical structure [86]. They have 2 benzene rings denoted by ring A and ring B connected to a third pyran ring that is ring C [87]. These phytochemicals play a major role in preventing the peroxidation of lipids by using processes such as electron transfer or chelation of metal ions [88]. The B ring that is present in the molecular structure of flavonoids engage a major role in the scavenging of free radicals. The B ring contains hydroxyl groups, which stabilize the free radical species, such as hydroxyl or peroxynitrite, by transferring either electrons or hydrogen atoms to them [89]. Flavonoids further prevent oxidative stress by chelating metal ions such as copper or ferric ions which stimulate the production of ROS in the body [89]. Different types of flavonoids exhibiting antioxidant activity include flavonols, flavones, isoflavone, and anthocyanidin and are found mainly in citrus fruits, tea, onion, berries, broccoli and soybean [86].

Stilbenes are a major sub-class of polyphenols present in the plants which also show antioxidant activity [90]. Stilbenes such as resveratrol help in preventing the oxidative stress to proteins and lipids and it also increases the activity of antioxidant enzymes such as GPx and SOD [91]. Phenolic acids such as salicylic acid, vanillic acid, caffeic acid also show significant antioxidant activity [85].

### 2.5. Polysterols

Polysterols are a subclass of sterols, which are a type of lipid characterized by a specific chemical structure containing a steroid nucleus [92]. These compounds naturally occur in plants and have gained recognition for their potential health benefits, particularly due to their antioxidant properties [93]. Polysterols possess the ability to scavenge free radicals and reduce oxidative stress within the body, thereby contributing to overall health and wellbeing [82]. One example of a polysterol compound with potent antioxidant activity is beta-sitosterol, which has been studied for its potential role in promoting cardiovascular health and supporting the immune system [94]. Another example is campesterol, which also exhibits antioxidant effects and may contribute to the prevention of chronic diseases associated with oxidative damage [95].

A comprehensive list of phytochemical classes and their representative antioxidant molecules, along with their high-altitude plant sources and their associated health benefits is shown in Table 1.

**Table 1:** List of phytochemical classes, along with representative molecules within each class having antioxidant property, their sources from high-altitude plants and their therapeutic properties against various oxidative stress associated diseases.

Phytochemical Class	Sub-Class	Representative Compounds	Chemical Formulae	PubChem ID	High Altitude Plant Source	Preventive Activity Against	Reference
Carotenoids	Carotenes	Alpha-carotene	C_40_H_56_	6419725	*Gentiana algida* Pall., *Rhododendron ferrugineum* L., *Ranunculus glacialis* L., *Saxifraga oppositifolia* L., *Primula hirsuta* All.	Cardiovascular diseases, type 2 diabetes, cancer, skin and eye diseases, ageing, inflammation	[96,97]
		Beta-carotene	C_40_H_56_	5280489			
		Lycopene	C_40_H_56_	446925			
		Phytoene	C_40_H_64_	5280784			
		Phytofluene	C_40_H_62_	6436722			
	Xanthophylls	Lutein	C_40_H_56_O_2_	5281243			
		Canthaxanthin	C_40_H_52_O_2_	5281227			
		Antheraxanthin	C_40_H_56_O_3_	5281223			
		Zeaxanthin	C_40_H_56_O_2_	5280899			
		β-cryptoxanthin	C_40_H_56_O	5281235			
		Astaxanthin	C_40_H_52_O_4_	5281224			
		Fucoxanthin	C_42_H_58_O_6_	5281239			
		Rubixanthin	C_40_H_56_O	5281252			
		Violaxanthin	C_40_H_56_O_4_	448438			
Vitamins	Ascorbic Acid		C_6_H_8_O_6_	54670067	*Vaccinium macrocarpon* Aiton. (Mountain cranberry), *Sorbus aucuparia* Poir., *Sorbus scopulina* Greene, *Juniperus recurva* Buch. -Ham. ex D. Don.	Age-related muscular degeneration, cataract, cardiovascular diseases, immunosuppression	[98,99]
	Tocopherols	Alpha-tocopherol	C_29_H_50_O_2_	14985		Cardiovascular diseases, cancer, obesity, diabetes	
		Beta-tocopherol	C_28_H_48_O_2_	6857447			
		Gama-tocopherol	C_28_H_48_O_2_	92729			
		Delta-tocopherol	C_27_H_46_O_2_	92094			
	Tocotrienols	Alpha-tocotrienol	C_29_H_44_O_2_	5282347			
Polyphenols	Flavonoids	Quercetin	C_15_H_10_O_7_	5280343	*Rhodiola rosea* L., *Vaccinium vitis-idaea* L., *Dipsacus fullonum* L., *Dipsacus sylvestris* Huds., *Juniperus recurva* Buch. -Ham. ex D. Don.	Obesity, neurodegenerative diseases, type 2 diabetes, and cardiovascular diseases	[100,101]
		Kaempferol	C_15_H_10_O_6_	5280863			
		Fisetin	C_15_H_10_O_6_	5281614			
		Isorhamnetin	C_16_H_12_O_7_	5281654			
		Myricetin	C_15_H_10_O_8_	5281672			
		Luteolin	C_15_H_10_O_6_	5280445			
		Apigenin	C_15_H_10_O_5_	5280443			
		Sinensetin	C_20_H_20_O_7_	145659			
		Isosinensetin	C_20_H_20_O_7_	632135			
		Nobiletin	C_21_H_22_O_8_	72344			
		Tangeretin	C_20_H_20_O_7_	68077			
		Galangin	C_15_H_10_O_5_	5281616			
		Chrysin	C_15_H_10_O_4_	5281607			
		Baicalin	C_21_H_18_O_11_	64982			
		Catechin	C_15_H_14_O_6_	9064			
		Epicatechin	C_15_H_14_O_6_	72276			
		Epicatechin gallate	C_22_H_18_O_10_	107905			
		Gallocatechin	C_15_H_14_O_7_	65084			
		Epigallocatechin	C_15_H_14_O_7_	72277			
		Epigallocatechin gallate	C_22_H_18_O_11_	65064			
		Daidzein	C_15_H_10_O_4_	5281708			
		Genistein	C_15_H_10_O_5_	5280961			
		Daidzin	C_21_H_20_O_9_	107971			
		Naringenin	C_15_H_12_O_5_	439246			
		Naringin	C_27_H_32_O_14_	442428			
		Hesperidin	C_28_H_34_O_15_	10621			
		Hesperetin	C_16_H_14_O_6_	72281			
		Eriodicytol	C_15_H_12_O_6_	11095			
		Pelargonidin	C_15_H_11_O_5_⁺	440832			
		Cyanidin	C_15_H_11_O_6_⁺	128861			
		Delphinidin	C_15_H_11_ClO_7_	68245			
		Peonidin	C_16_H_13_O_6_⁺	441773			
		Petunidin	C_16_H_13_O_7_⁺	441774			
		Malvidin	C_17_H_15_O_7_⁺	159287			
	Stilbenes	Resveratrol	C_14_H_12_O_3_	445154			
		Pinosylvin	C_14_H_12_O_2_	5280457			
		Piceatannol	C_14_H_12_O_4_	667639			
		Pterostilbene	C_16_H_16_O_3_	5281727			
		Rhapontigenin	C_15_H_14_O_4_	5320954			
		Isorhapontigenin	C_15_H_14_O_4_	5318650			
	Phenolic acids	Salicylic acid	C_7_H_6_O_3_	338			
		Hydroxybenzoic acid	C_7_H_6_O_3_	135			
		Protocatechuic acid	C_7_H_6_O_4_	72			
		Gallic acid	C_7_H_6_O_5_	370			
		Syringic acid	C_9_H_10_O_5_	10742			
		Vanillic acid	C_8_H_8_O_4_	8468			
		Gentisic acid	C_7_H_6_O_4_	3469			
		Coumaric acid	C_9_H_6_O_2_	323			
Phytosterols		Campesterol	C_28_H_48_O	173183	*Rhodiola* spp., *Dipsacus* spp., *Juniperus* spp.	Elevated cholesterol level, inflammation, oxidative stress, immunosuppression.	[102,103]
		Sitosterol	C_29_H_50_O	222284			
		Stigmasterol	C_29_H_48_O	5280794			
		Campestanol	C_28_H_50_O	119394			
		Stigmastanol	C_29_H_52_O	241572			

## 3. Role of Phytochemical Antioxidants in Mitigating Major Lifestyle-Associated Oxidative Stress-Induced Health Disorders

### 3.1. Cardiovascular Diseases

**Figure 4:**
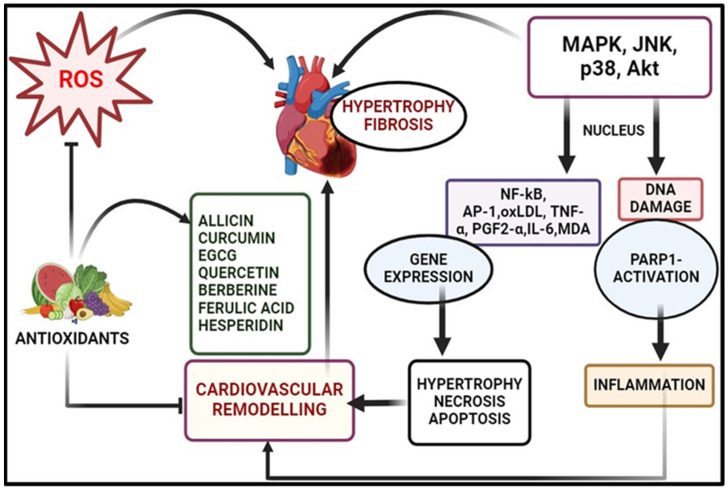
Oxidative stress-induced cardiovascular diseases and modulation via phytochemical antioxidants (the illustration was created using BioRender at www.biorender.com). [MAPK: mitogen-activated protein kinase; ROS: reactive oxygen species; JNK: c-Jun N-terminal kinase; p38: p38 mitogen-activated protein kinase; Akt: protein kinase B (PKB); NF-κB: nuclear factor kappa-light-chain-enhancer of activated B cells; AP-1: activator protein 1; oxLDL: oxidized low-density lipoprotein; TBF-α: tumour necrosis factor alpha; PGF2-α: prostaglandin F2 alpha; IL-6: interleukin 6; MDA: malondialdehyde; PARP-1: poly (ADP-ribose) polymerase 1].

Cardiovascular disease (CVD), the leading cause of global mortality, is intricately linked to oxidative damage, with ROS orchestrating various deleterious effects [104]. As discussed in the section regarding cardiovascular diseases, elevated ROS levels diminish nitric oxide availability, inducing vasoconstriction and hypertension, while also disrupting myocardial calcium handling, leading to arrhythmias and cardiac remodelling via hypertrophic signalling and apoptosis [105,106] (Figure 4). Chronic oxidative stress in heart failure triggers cardio myocyte apoptosis, fibrosis, and mitochondrial dysfunction, perpetuating myocardial damage and dysfunction through pro-inflammatory cytokine activation, fibrotic growth factor release, and impaired calcium homeostasis. Atrial fibrillation (AF) is the most common cardiac arrhythmia, fuelled by oxidative stress-induced atrial remodelling and inflammation, promoting structural changes and fibrosis, which create a substrate for atrial fibrillation [107].

In the relentless pursuit to mitigate oxidative damage in cardiovascular tissue, there has arisen a growing interest in the utilization of medicinal plants as natural antioxidants [108]. The bioactive components derived from these botanical sources, encompassing polyphenols and polysaccharides commonly found in traditional herbal medicine, hold promise in combatting oxidative stress and its associated cardiovascular disorders [109]. Table 2 delineates the myriad plant bioactive compounds targeting oxidative stress pathways and related cardiovascular diseases. As free radicals instigate a chain reaction of oxidative damage within cardiovascular tissues [19], the active constituents found in medicinal plants serve as potent scavengers, blocking this detrimental process through both direct and indirect mechanisms [110].

One important example is curcumin, which is derived from the turmeric plant and is renowned for its anti-inflammatory and antioxidant properties. Curcumin exerts antioxidant effects by directly scavenging free radicals and upregulating endogenous antioxidant enzymes [111]. It also inhibits inflammatory pathways, such as NF-κB pathway, thereby mitigating inflammation and oxidative stress in cardiovascular tissues [111]. Epigallocatechin gallate (EGCG), found in tea, is renowned for its potent antioxidant and cardioprotective effects. EGCG modulates signalling pathways involved in oxidative stress and inflammation, such as the MAPK and PI3K/Akt pathways, thereby protecting against cardiovascular diseases [112]. Quercetin, abundant in various fruits, vegetables, and teas, functions as a free radical scavenger, inhibits lipid peroxidation, and enhances the activity of antioxidant enzymes like SOD and CAT. Furthermore, it modulates inflammatory pathways, including NF-κB and COX, thereby mitigating oxidative stress and inflammation in cardiovascular tissues [113].

Given their favourable safety profile and multifaceted antioxidative properties, the exploration and integration of plant-derived phytochemical antioxidants into clinical practice hold tremendous potential for ameliorating oxidative stress in the management of cardiovascular disorders [114].

**Table 2:** Phytochemicals, along with their high-altitude plant sources, are reported to mitigate oxidative stress-induced cardiovascular diseases [108].

Phytochemical	Plant	Chemical Structure	Treatment	Mechanism of Action	Reference
Allicin	*Allium humile* Kunth	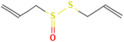	Hypertension	Inhibits the formation of LPO and MDA	[108,115]
Berberine	*Berberis aristata* DC.	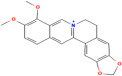	Hypertension	Reduces O_2_ and H_2_O_2_ levels	[116]
Delphinidin-3-glucoside	*Vaccinium myrtillus* L.	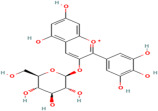	Coronary heart disease, ischemia-reperfusion injury	Inhibits caspase-3, bax, and ap-JNK expression	[117,118]
Gastrodin	*Gastrodia elata* Blume.	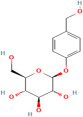	Heart failure	Regulates AMPK, Akt, mTOR, and Bcl-2	[119]
Gypenoside	*Gynostemma pentaphyllum* Thunb.	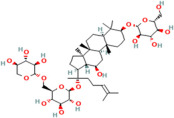	Acute myocardial infarction	Regulates the PI3K/Akt/mTOR signalling pathway	[120,121]
Matrine	*Sophora* *flavescens* Aiton.	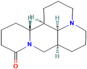	Arrhythmia	Increases production of SOD	[122,123,124]
Orientin	*Millettia nitida* Benth.	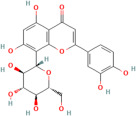	Coronary heart disease, atherosclerosis	Reduces ROS	[125,126,127]
Paeonol	*Paeonia* *suffruticosa* Andrews	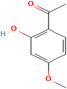	Arrhythmia, coronary heart disease	Inhibits free radical reaction	[122,128]
Polysaccharides	*Astragalus propinquus* Schischk.		Coronary heart disease, acute myocardial infarction	Inhibits the expression of NOX	[129]
Quercetin	*Dendrobium nobile* Lindl.	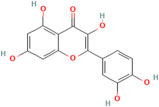	Acute myocardial infarction, ischemia Reperfusion	Reduce ROS	[130]
Tanshinone II-A	*Salvia* *miltiorrhiza* Bunge.	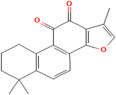	Coronary heart disease, acute myocardial infarction	Regulates Nrf2/ARE/HO-1 and TGF-beta1/signal transduction	[131,132]
Tetramethylpyrazine	*Ligusticum chuanxiong*	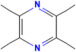	Heart failure, coronary heart disease	Increases the activity of SOD, CAT and GSH-Px	[133,134]

[LPO: lipid peroxidation; MDA: malondialdehyde; O_2_: oxygen; H_2_O_2_: hydrogen peroxide; bax: Bcl-2-associated X protein; ap-JNK: activator protein-1 c-Jun N-terminal kinase; AMPK: AMP-activated protein kinase; Akt: protein kinase B; mTOR: mechanistic target of rapamycin; Bcl-2: B-cell lymphoma 2; *Bad*: Bcl-2-associated death promoter; PI3K: phosphoinositide 3-kinase; SOD: superoxide dismutase; ROS: reactive oxygen species; NOX: NADPH oxidase; Nrf2: nuclear factor erythroid 2-related factor 2; ARE: antioxidant response element; HO-1: heme oxygenase 1; TGF-beta1: transforming growth factor beta 1; CAT: catalase; GSH-Px: glutathione peroxidase].

### 3.2. Neurodegenerative Disorders

**Figure 5:**
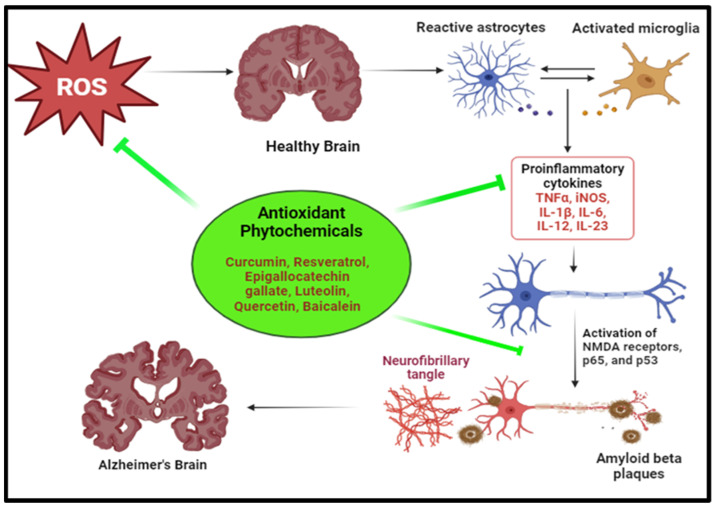
Oxidative stress-induced neurodegenerative disease pathology and modulation by antioxidant phytochemicals. (the illustration was created using BioRender at www.biorender.com). [TNFα: tumour necrosis factor alpha, iNOS: inducible nitric oxide synthase, IL-1β: interleukin-1 beta, IL-6: interleukin-6, IL-12: interleukin-12, IL-23: interleukin-23, NMDA: N-methyl-D-aspartate, p65: RelA (a subunit of the NF-κB transcription factor), p53: tumour protein p53].

Neurodegenerative disorders involve the loss of functional capacity and eventual dysfunction or death of neuronal cells in the brain [135]. Diseases like Parkinson’s and Alzheimer’s are characterized by neurodegeneration, and oxidative stress plays a major role in their pathogenesis [136]. The high level of ROS generation and low antioxidant levels in brain cells make them susceptible to oxidative damage, which alters the function of lipids, DNA and proteins, contributing to neurodegeneration (Figure 5) [137,138].

In Alzheimer’s disease, ROS stimulate the cleavage of amyloid precursor protein (APP), enhancing the production of Aβ peptides which aggregates to form toxic Aβ plaques. [139]. During oxidative stress, ROS induces activation of kinases and inhibition of phosphatases leading dysregulate *tau* phosphorylation dynamics which destabilizes microtubules and leads to their aggregation into neurofibrillary tangles [140]. ROS overwhelm the endogenous antioxidant defence system, which amplifies oxidative damage and potentiates neuronal vulnerability. The activation of microglia initiates an inflammatory cascade, which starts a pro-inflammatory cytokine release and causes exacerbate neuroinflammation, contributing to neuronal dysfunction and degeneration [32]. Sequential lipid peroxidation generates breakdown products like 4-hydroxy-2,3-nonenal (HNE), elevated levels of which, in brain tissues, is indicative of Alzheimer’s disease [141,142].

Parkinson’s disease, the second most common neurodegenerative disorder in elderly individuals, on the other hand, primarily affects the motor functions of the body, leading to noticeable movement disorders. OS promotes the misfolding of α-synuclein protein, which aggregates to form Lewy bodies. PD is linked with increased levels of HNE in brain tissues. Increased levels of 8-hydroxyguanine and 8-hydroxy-2-deoxyguanosine, resulting from oxidative damage to DNA base pairs, are also indicative of Parkinson’s disease [33,139].

Several potent phytochemicals have shown potential in combating neurodegenerative diseases, offering avenues for novel therapeutic interventions [143] (Table 3). For Alzheimer’s disease, compounds like curcumin, found in turmeric, exhibit anti-inflammatory and antioxidant properties, inhibiting the formation of beta-amyloid plaques and reducing neuroinflammation [144]. 

Resveratrol, abundant in red grapes and berries, demonstrates neuroprotective effects by modulating signalling pathways involved in neuronal survival and reducing oxidative stress [145]. Similarly, flavonoids, such as EGCG found in green tea, and quercetin, which is abundant in onions and apples, possess neuroprotective properties by scavenging free radicals and inhibiting neuroinflammation [146]. 

In Parkinson’s disease, phytochemicals, like polyphenols, particularly found in berries, cocoa, and grapes, exhibit neuroprotective effects by enhancing mitochondrial function, reducing oxidative stress, and inhibiting alpha-synuclein aggregation [147]. Additionally, compounds, like sulforaphane, present in cruciferous vegetables, activate cellular defence mechanisms against oxidative stress and inflammation, potentially mitigating neuronal damage in Parkinson’s disease [148]. 

Phytochemical compounds have also been found to decrease the risk of 4-hydroxy-2,3-nonenal (HNE) aggregation, a reactive aldehyde produced during oxidative stress and implicated in various neurodegenerative diseases. For instance, polyphenolic compounds, such as curcumin, found in turmeric, and resveratrol, abundant in red grapes and berries, have been shown to inhibit HNE-induced protein aggregation and lipid peroxidation [149]. Additionally, flavonoids, like EGCG from tea and quercetin from onions and apples, have demonstrated protective effects against HNE-induced toxicity by modulating cellular signalling pathways and enhancing antioxidant defences. These compounds possess strong antioxidant properties, scavenging free radicals and mitigating oxidative damage and thereby reducing the formation of HNE adducts and subsequent aggregation [150]. 

**Table 3:** Phytochemicals, along with their high-altitude plant sources, reported to mitigate oxidative stress-induced neurodegenerative disorders [151].

Phytochemicals	Plant	Structure	Mode of Action	Reference
1,8-Cineole	*Salvia officinalis* L.	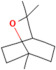	Selectively suppresses NF- κB and activation of pro-inflammatory gene expression and cytokine production, enhances neurogenesis	[152]
Asiatic acid	*Centella asiatica* (L.) urban	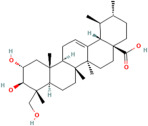	Inhibits pro-inflammatory cytokines and inflammatory pathway and promotes neurogenesis	[153,154]
Asiaticoside	*Centella asiatica* (L.) urban	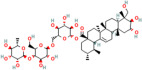	Inhibits pro-inflammatory cytokines	[155,156]
Bacoside A	*Bacopa monniera* (L.) Pennel	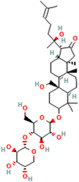	Reduces oxidative stress-induced neuronal damage, enhances cholinergic neurotransmission, improves cognitive function, inhibits pro-inflammatory cytokines, inhibits amyloid-beta (Aβ) peptide aggregation, and promotes synaptic remodelling	[157,158]
Baohuoside I	*Centella asiatica* (L.) urban	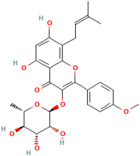	Promotes the antioxidant activity of essential enzyme such as SOD, CAT and GSH-Px.	[159]
Betulic acid	*Centella asiatica* (L.) urban	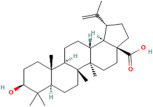	Inhibiting pro-inflammatory cytokines and signalling pathways and promotes neurotrophic factor BDNF expression contributing to overall brain health	[160]
Borneol	*Salvia officinalis* L.	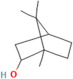	Exhibits antioxidant properties and suppresses pro-inflammatory cytokine production	[161]
Brahmic acid	*Centella asiatica* (L.) urban	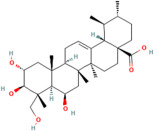	Promotes neurogenesis; modulates neurotransmitter levels, including acetylcholine, serotonin, and dopamine; and reduces the production of pro-inflammatory cytokines	[155]
Camphor	*Salvia officinalis* L.	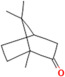	Exhibits antioxidant properties and suppresses NF-κB activation and pro-inflammatory cytokine production	[162]
Caryophyllene	*Salvia officinalis* L.	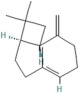	Demonstrates anti-inflammatory activity, modulates neurotransmitter systems and enhances neurogenesis	[163]
Herpestine	*Bacopa monniera* (L.) Pennel	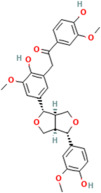	Enhances neuronal synthesis, increases kinase activity, and restores synaptic activity and nerve impulse transmission	[164]
Linalool	*Salvia officinalis* L.	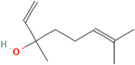	Scavenges free radicals, suppresses NF-κB activation and pro-inflammatory cytokine production, modulates neurotransmitter systems and enhances neurogenesis	[152]
Luteolin	*Picrorhiza scrophulariiflora* Pennell.	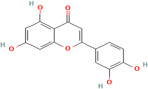	Reduces neuroinflammation, promotes expression of brain-derived neurotrophic factor (BNDF) and modulates neurotransmitter systems, such as dopamine and serotonin	[165]
Madecassic acid	*Centella asiatica* (L.) urban	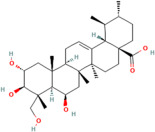	Inhibits pro-inflammatory cytokines and signalling pathways and promotes neurotrophic factors’ BDNF expression	[160,166]
Picroside II	*Picrorhiza scrophulariiflora* Pennell.	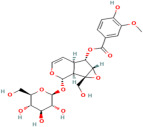	Inhibits neuronal apoptosis	[167]

[NF-κB: Nuclear factor kappa-light-chain-enhancer of activated B cells; SOD: superoxide dismutase; CAT: catalase; GSH-Px: glutathione peroxidase; BDNF: brain-derived neurotrophic factor].

### 3.3. Metabolic Disorders: Diabetes and Obesity

**Figure 6:**
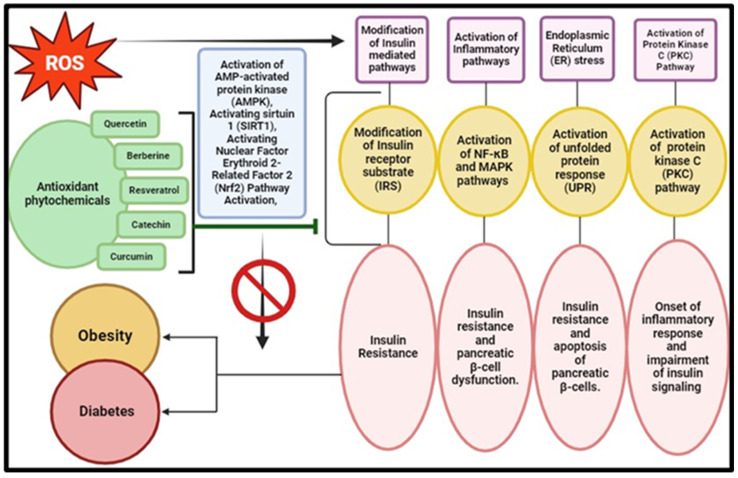
Antioxidant phytochemicals and modulation of oxidative stress-induced metabolic disorders (obesity and diabetes) (the illustration was created using BioRender at www.biorender.com). [AMPK: AMP-activated protein kinase; SIRT1: sirtuin 1; Nrf2: nuclear factor erythroid 2-related factor; 2NF-κB: nuclear factor kappa-light-chain-enhancer of activated B cells; MAPK: mitogen-activated protein kinase; IRS: insulin receptor substrate; UPR: unfolded protein response; PKC: protein kinase C].

Metabolic disorders, including obesity and diabetes, are closely linked with the generation of ROS in the body [8]. Studies have shown a positive correlation between decreased levels of high-density lipoproteins (HDLs) and increased levels of low-density lipoproteins (LDLs) with oxidative stress. Lower levels of HDLs result in dysfunctional antioxidant defence mechanisms, leading to elevated oxidative stress (Figure 6) [168,169]. OS is also implicated in obesity as excessive ROS production acts as a trigger for abnormal amplification and enlargement of pre-adipocytes and adipocytes. This abnormal adipose cell growth leads to adipogenesis, a fundamental factor in obesity [37]. Improper dietary patterns, including high carbohydrate and high-fat diets, can increase oxidative stress in the body, contributing to obesity [170].

Diabetes, on the other hand, characterized by high glucose levels and decreased insulin sensitivity, is another metabolic disorder linked to oxidative stress [36]. Mitochondrial dysfunction resulting from oxidative stress contributes significantly to insulin resistance, impairing insulin responses and leading to abnormal glucose levels [171]. Oxidative radicals also promote apoptosis in pancreatic beta-cells, modifying cell cycle regulators and contributing to the diabetes development [12]. In type 2 diabetes, islet inflammation causing pancreatic β cell dysfunction underscores inflammation’s significance [172]. Concurrently, oxidative stress in people with diabetes and obesity plays a major role in causing cardiovascular associated complications as well [173].

Plant products have been gaining attention for the potential mitigation of metabolic disorders by modulating proinflammatory cytokines and ROS. Methanolic extracts from *Capparis spinosa* L. leaves show in vitro anti-inflammatory effects, inhibiting membrane destabilization, and exerting anti-inflammatory effects in murine models [174]. Plant secondary metabolites like carotenoids and alkaloids induce an anti-inflammatory response by suppressing IL-17 and inducing IL-4 gene expression [175]. With concerns about synthetic antioxidants’ long-term safety, there is rising demand for natural antioxidants to mitigate oxidative stress-related diseases [176]. Recognized as rich in essential antioxidants, plants are increasingly viewed as functional ingredients promoting health. Plant-derived products, including phytochemicals, emerge as a valuable natural source of anti-inflammatory agents with potential therapeutic implications for metabolic disorders (Table 4). For instance, curcumin, resveratrol, quercetin, epigallocatechin gallate, berberine, and alpha-lipoic acid have garnered significant attention for their potential in mitigating oxidative stress-related metabolic dysregulations [177]. Curcumin exerts its effects through NF-κB pathway inhibition, activation of the Nrf2 pathway, and modulation of insulin signalling, thereby offering therapeutic benefits in diabetes, obesity, and cardiovascular diseases [178]. Similarly, resveratrol activates sirtuin 1 (SIRT1), possesses antioxidant activity, and activates AMP-activated protein kinase (AMPK), contributing to its medicinal properties against metabolic disorders [179]. Quercetin scavenges free radicals, modulates inflammatory pathways, and enhances mitochondrial function, making it beneficial for metabolic health. EGCG exhibits antioxidant activity, regulates insulin signalling, and modulates adipocyte function, thereby improving metabolic parameters in various disorders [180]. Berberine activates AMPK, modulates gut microbiota, and inhibits inflammatory pathways, offering therapeutic potential in metabolic disorders [181]. Alpha-lipoic acid exerts antioxidant effects, regulates mitochondrial function, and modulates insulin signalling, contributing to its efficacy against metabolic dysregulations [182]. Overall, understanding the mechanistic insights into these plant bioactive compounds is crucial for developing targeted strategies to combat oxidative stress-related metabolic disorders and improve public health outcomes.

**Table 4:** Phytochemicals, along with their high-altitude plant sources, as treatment options against metabolic disorders [183].

Phytochemical	Plant	Chemical Structure	Mode of Action	Reference
Anthocyanin	*Aristotelia chilensis* (Molina) Stuntz	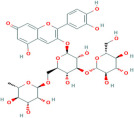	Inhibits synthesis of the pro-inflammatory cytokines, TNF-α and IL-6, further reducing inflammation associated with diabetes and obesity, and modulates the NF-κB signalling pathway, leading to decreased expression of inflammatory mediators	[184]
Ascorbic acid	Rosehips produced by *Rosa pendulina* L.	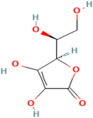	Enhances insulin sensitivity, facilitating the uptake of glucose into cells; reduces risk of hyperglycaemia; and modulates lipid metabolism by reducing lipid peroxidation and inhibiting fatty acid synthesis, which prevents dyslipidemia	[185,186]
Caffeine	*Ilex guayusa* Loes.	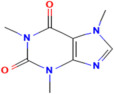	Stimulates lipolysis and thermogenesis, caffeine may help reduce circulating levels of LDL cholesterol and triglycerides, thereby preventing the development of atherosclerotic plaques	[187]
Niazirin	*Moringa oleifera* Lam.	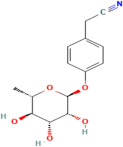	Helps regulate lipid metabolism, reducing the level of triglyceride and LDL cholesterol while increasing the production of HDL cholesterol; modulates lipid metabolism and helps prevent the formation of atherosclerotic plaques; and maintains vascular health in diabetic individuals.	[188,189]
Proanthocyanidins	*Vitis vinifera* L.	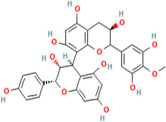	Promotes endothelial NO production, leading to vasodilation and improved blood flow; inhibits endothelial cell apoptosis and preserve vascular homeostasis; prevents formation of atherosclerotic plaques; and maintains cardiovascular health	[190]
Phenolic acids (Protocatechuic acid) and saponins	*Androsace umbellata* (Lour.) Merr.	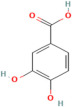	Promotes the production of serum antioxidant enzymes, upregulates the expression of hepatic antioxidant genes, and inhibits the NF-κB signalling pathway, leading to the decreased expression of inflammatory mediators	[191,192]

[TNF-α: tumour necrosis factor alpha; IL-6: interleukin 6; NF-κB: nuclear factor kappa-light-chain-enhancer of activated B cells; NO: nitric oxide; LDL: low-density lipoprotein; HDL: high-density lipoprotein].

## 4. High-Altitude Medicinal Plants: Bulk Producers of Antioxidants

Plants that thrive in high-altitude environments face numerous challenges due to extreme environmental conditions, such as low carbon dioxide and oxygen levels, intense mutagenic radiation, and drastic temperature fluctuations. These factors create a harsh survival environment for plants. ROS production is heightened in these plants, leading to cellular damage and impairing photosynthesis. In response to these conditions, plants have developed adaptive mechanisms to counteract the negative effects of oxidative stress caused by ROS [193].

High-altitude plants have evolved the ability to synthesize secondary metabolites in large quantities. These metabolites, including flavonoids, phenols, tannins, and other compounds, serve as antioxidants within the plants [47]. By accumulating these secondary metabolites, plants can adapt to the extreme environmental conditions and mitigate the harmful effects of ROS-induced oxidative damage. This defence mechanism helps these plants maintain their cellular integrity and sustain their growth and survival in such harsh environments [194].

### 4.1. Environmental Factors Influencing Antioxidant Production in High-Altitude Medicinal Plants

High-altitude regions, characterized by unique environmental conditions, present challenges as well as opportunities for plant life. The synthesis of antioxidant phytochemicals in high-altitude medicinal plants is influenced by several environmental factors:

#### 4.1.1. Solar Radiation Intensity and Ultraviolet (UV) Exposure

High-altitude regions often experience increased solar radiation due to reduced atmospheric filtration. Elevated UV radiation levels can lead to oxidative stress in plant tissues by generating ROS. Plants respond by activating antioxidant defence mechanisms modulated by flavonoids such as the production of quercetin, kaempferol, catechins and others to scavenge ROS and protect cellular components [195].

#### 4.1.2. Temperature Fluctuations

High-altitude environments exhibit significant diurnal temperature variations, including cold nights and warm days. Temperature fluctuations can disrupt cellular homeostasis and induce oxidative stress. High-altitude plants adapt by synthesizing antioxidant compounds such as chlorogenic acid, a phenolic compound, to mitigate temperature-induced oxidative damage and maintain cellular integrity [96].

#### 4.1.3. Low Oxygen Levels (Hypoxia)

Reduced atmospheric pressure at higher elevations results in lower oxygen levels, leading to hypoxic conditions. Hypoxia-induced oxidative stress can occur due to impaired mitochondrial function and increased ROS production. High-altitude plants enhance the production of alkaloid antioxidants such as berberine to counteract hypoxia-induced oxidative damage [96].

#### 4.1.4. Water Scarcity and Drought Stress

Water availability in high-altitude regions can be limited, particularly in arid or semi-arid environments. Drought stress disrupts cellular hydration and photosynthetic processes, triggering oxidative stress. High-altitude plants accumulate osmo-protectants, such as proline and antioxidants, such as flavonoids to mitigate water stress-induced oxidative damage and maintain cellular hydration [196].

#### 4.1.5. Soil Composition and Nutrient Availability

High-altitude soils often exhibit low nutrient availability, high acidity, and metal-rich compositions. Adverse soil conditions can exacerbate oxidative stress in plants by limiting nutrient uptake and promoting metal-induced ROS generation. High-altitude plants produce metal chelators that bind and detoxify heavy metals present in the soil, reducing metal-induced oxidative stress. Polyphenols scavenge ROS and regulate nutrient uptake, contributing to antioxidant defence and nutrient homeostasis [197].

#### 4.1.6. Altitude-Dependent Factors

Altitude-specific variables, including atmospheric pressure, humidity, and air pollution, influence antioxidant production in high-altitude plants. Changes in atmospheric pressure and humidity modulate plant metabolism and ROS production, while air pollutants like ozone and nitrogen oxides contribute to oxidative stress. High-altitude plants adjust their antioxidant defences, such as the production of terpenoids, which exhibit adaptogenic properties, enhancing plant resilience to altitude-dependent stressors like changes in atmospheric pressure and humidity. Anthocyanins act as antioxidants and UV protectants, shielding plant tissues from oxidative damage and UV radiation at high altitudes [96].

### 4.2. High-Altitude Plants and Their Antioxidant Potential

Plants have held a significant role in the field of medicine since ancient times [198]. Various plant species, such as Tulsi and Neem, have been recognized for their beneficial effects on human health, functioning as antibacterial, anti-inflammatory, and antioxidant agents [199]. In particular, certain plants found in the high-altitude regions possess unique properties and produce phytochemicals and essential oils, rich in phenolic compounds and flavonoids etc. These phytochemicals have the ability to scavenge free radicals through various mechanisms, such as electron donation, hydrogen atom donation, acting as reducing agents, or chelation of metal ions [82]. By employing these strategies, they effectively neutralize harmful free radicals, thereby providing antioxidant protection. These natural compounds hold great promise in the field of drug discovery, as they serve as botanical leads for the development of novel therapeutic agents. The following section describes selected high-altitude medicinal plants along with their antioxidant potential.

#### 4.2.1. *Saussurea lappa* (Decne.) C. B. Clarke

*Saussurea lappa* (Decne.) C. B. Clarke is a medicinal plant that belongs to the Asteraceae family and is predominantly found at high altitudes, ranging from 2500 to 3500 m above mean sea level, primarily in the Himalayan region [200]. It is commonly referred to as ‘Costus’ and has garnered significant attention due to its extensive medicinal applications. Notably, this plant is enriched with essential vitamins, including vitamin B12, vitamin B2, vitamin A, as well as vital minerals such as calcium, iron, and zinc [201].

A distinctive feature of *Costus* is the presence of a phytochemical called costunolide, which is primarily found in its roots [201]. Costunolide exhibits remarkable antioxidant activity, which has been attributed to its ability to counteract the development of cancer [202]. The compound contains N-acetylcysteine, which plays a pivotal role in neutralizing ROS by facilitating the production of key enzymes like SOD and CAT [203]. These aid in the detoxification of harmful free radicals, thereby contributing to the plant’s antioxidant defence system. Through its antioxidant properties, *Costus* holds potential as a therapeutic agent in the prevention and management of various diseases [204].

#### 4.2.2. *Arnebia benthamii* (Wall. ex G. Don) I. M. Johnst.

*Arnebia*, scientifically known as *Arnebia benthamii* (Wall. ex G. Don) I. M. Johnst., is a highly valued medicinal plant belonging to the Boraginaceae family. It thrives in high-altitude Himalayan regions, specifically ranging from 3000 to 3900 m above mean sea level [205]. However, it is important to note that this plant has been classified as a critically endangered species in the Northwestern Himalayas by the International Union for Conservation of Nature (IUCN) [206]. Himalayan *Arnebia* possess various phytochemicals, including a prominent compound called shikonin [207]. Shikonin plays a vital role in preventing oxidative DNA damage through its free radical scavenging mechanism. As a quinone derivative, shikonin acts as a potent antioxidant, effectively thwarting lipid peroxidation and DNA damage by neutralizing free radicals and reducing ferrous ions [82].

The antioxidative properties of shikonin contribute to the overall preservation of cellular integrity, providing a protective shield against oxidative stress. The presence of shikonin in Himalayan *Arnebia* underscores its medicinal significance and potential therapeutic applications [208]. Studies have highlighted the antioxidant capabilities of this plant, shedding light on its role in preventing oxidative damage and maintaining cellular health [209,210].

#### 4.2.3. *Pinus nigra* Aiton, Hort. Kew. [W. Aiton]

Belonging to the Pinaceae family, this particular plant species thrives in the high-altitude regions (2000 m above mean sea level) of the Toros mountains and holds a great significance in combatting oxidative stress-induced damage [211]. This plant abundantly produces phenols and flavonoids, which are extremely efficient in neutralizing several free radical species, including hydrogen peroxides and superoxide free radical species, including superoxide radicals and hydrogen peroxide. This antioxidant activity is facilitated through multiple mechanisms, such as chelation of metal ions, free radical scavenging and the reduction of ferrous ions [212].

The presence of phenols and flavonoids in this plant demonstrates its adaptation to cope with the challenging environmental conditions it encounters. By effectively neutralizing free radicals, these compounds help protect the plant’s cellular components from oxidative damage and maintain their functionality. Studies have highlighted the antioxidative properties of this plant, shedding light on its potential role in preventing oxidative stress-related disorders [213].

#### 4.2.4. *Cedrus deodara* (Roxb. ex D. Don) G. Don

*Cedrus deodara*, also known as the Deodar cedar, is a significant plant that has been used in Ayurveda for its medicinal benefits [214]. It is an evergreen plant found at high altitudes, specifically around 3000—3300 m above mean sea level [214]. Belonging to the family Pinaceae, this plant contains phytochemicals, such as ‘Metairesinol,’ which exhibit antioxidant activity [215]. These phytochemicals help in inhibiting oxidative stress by chelating metal ions or transferring hydrogen atoms to free radical species [212].

#### 4.2.5. *Podophyllum hexandrum* Royle

*Podophyllum hexandrum*, also known as Himalayan May apple, is found at an altitude of around 3000—3500 m above mean sea level [216]. It belongs to the Berberidaceae family and exhibits high antioxidant activity due to phytochemicals, such as podophyllotoxin, present in its rhizome, leaves, and other parts [216]. The extracts of this plant are capable of neutralizing hydrogen peroxide and superoxide radicals, thus preventing lipid peroxidation, and also stimulate the activity of antioxidant enzymes [217].

#### 4.2.6. *Valeriana jatamansi* D. Don

*Valeriana jatamansi*, known as Mushkibala in Hindi, is a high-altitude medicinal plant found at an altitude of around 3000 m above mean sea level [218]. It belongs to the Valerianaceae family and possesses antiseptic and antioxidant properties [218]. It contains a class of terpenoids called valepotriates, which are responsible for its medicinal applications [219]. The rhizome of *Valeriana jatamansi* contains phenols and flavonoids, which exhibit antioxidant activity by donating hydrogen atoms or quenching singlet oxygen species. It can also chelate certain metal ions, thereby inhibiting the generation of ROS [220].

#### 4.2.7. *Berberis aristata* DC.

*Berberis aristata*, also known as Daru Haldhi, is a Himalayan shrub found at an altitude of around 2000—3000 m above sea level. It is primarily found in the areas of Himachal Pradesh, Nepal, and Sri Lanka [221]. *Berberis aristata* possesses antioxidant potential attributed to certain protoberberines present in its root and shoot extracts. These compounds contribute to the neutralization of ROS, reducing the risk of oxidative stress-related issues such as hepatic damage [222].

#### 4.2.8. *Pedicularis longiflora* Rudolph

*Pedicularis longiflora* is a plant widely found in the Himalayan regions of Ladakh, Jammu and Kashmir, at an altitude of approximately 2700 m above mean sea level [223]. This plant is valued for its medicinal properties, particularly its antioxidant and anti-inflammatory effects [224]. It contains phytochemicals such as flavonoids and phenols, which reduce lipid peroxidation by scavenging superoxide radicals. Moreover, *Pedicularis longiflora* enhances the activity of CAT and SOD, further contributing to its antioxidant activity [224].

#### 4.2.9. *Aconitum heterophyllum* Wall. ex Royle

*Aconitum heterophyllum*, also known as Indian aconite or Atees, is an Ayurvedic medicinal plant native to the Himalayan region, including Jammu and Kashmir, Nepal, Sikkim, and Uttarakhand, at altitudes ranging from 2500 to 4000 m above mean sea level [225]. It belongs to the Ranunculaceae family. The roots, stems, and leaves of this plant contain alkaloids and flavonoids, which play a crucial role in detoxifying ROS within the body [226]. These compounds contribute to the prevention of gastrointestinal problems such as liver inflammation [227].

### 4.3. Underutilization of High-Altitude Medicinal Plants

High-altitude regions, defined as areas above 1500 m (4900 feet) elevation, encompass diverse ecosystems ranging from alpine meadows to snow-capped peaks. These regions are home to a rich array of medicinal plants that have been traditionally used by indigenous communities for centuries to treat various ailments [228]. The harsh environmental conditions of high-altitude environments, including intense solar radiation, extreme temperatures, and oxidative stress, have driven the evolution of plants towards unique biochemical compositions and pharmacological properties. For instance, one example of a high-altitude medicinal plant with potent antioxidant properties is *Rhodiola rosea*, also known as golden root or arctic root. Indigenous to mountainous regions of Europe and Asia, *Rhodiola rosea* has been traditionally used to increase resistance to physical and environmental stress, enhance mental performance, and promote longevity. Studies have attributed its adaptogenic and antioxidant effects to bioactive compounds, including salidroside, rosavin, and flavonoids, which scavenge free radicals, reduce oxidative damage, and modulate stress-responsive pathways [14]. Similarly, *Berberis aristata*, a high-altitude plant native to the Himalayas, is valued for its medicinal properties, including its antioxidant, anti-inflammatory, and hepatoprotective effects. *Berberis aristata* contains bioactive alkaloids, such as berberine, palmatine, and berbamine, which exhibit potent antioxidant activity by neutralizing ROS, inhibiting lipid peroxidation, and enhancing cellular antioxidant enzymes [229].

However, despite their immense potential therapeutic benefits, a large variety of these plants, and the products they produce, remain largely underutilized in modern medicine. Several factors contribute to this underutilization [9]. Firstly, there is a lack of comprehensive scientific research exploring the antioxidant potential of high-altitude medicinal plants. Limited funding and resources are allocated to studying plants in remote mountainous regions, making it difficult to gather robust scientific evidence to support their medicinal properties. As a result, many of these plants remain overlooked in pharmaceutical and nutraceutical industries. Additionally, challenges in accessing high-altitude environments pose logistical difficulties for researchers. Harsh terrain, extreme weather conditions, and limited infrastructure make it challenging to conduct field studies and collect plant samples. This impedes efforts to characterize the bioactive compounds and pharmacological activities of high-altitude medicinal plants [230].

Furthermore, traditional knowledge of these plants is at risk of being lost as the young population inhabiting mountainous regions tends to migrate to urban areas and adopt modern lifestyles. The decline of indigenous knowledge and traditional healing practices contributes to the under appreciation of high-altitude medicinal plants in mainstream healthcare systems [231]. This necessitates the compilation of knowledge of important medicinal plants thriving in high-altitude regions, along with their reported bioactive compounds and their reported medicinal applications. Table 5 provides a comprehensive compilation of 168 such plants of medicinal value, which are able to survive in high-altitude regions, along with their ethnopharmacological applications. A large majority of these plants are unexplored and have not been utilized to their full potential. The comprehensive detailed analysis of their phytochemicals could act as starting point for the exploration of their potential to mitigate oxidative stress-related disorders.

**Table 5:** List of high-altitude medicinal plants, along with their reported bioactive compounds and their pharmacological properties.

S. No.	Plant Name	Plant Family	Altitude (m above m.s.l.)	Parts Used	Principle Bioactive Compound	Pharmacological Activity	Reference
1.	*Allium humile* Kunth	Amaryllidaceae	3200–4500	Whole plant	Allicin	Antioxidant	[232]
2.	*Allium semenovii* Regel.		2000–3000	Whole plant	Alliin	Antioxidant	[233]
3.	*Allium stoliczki* Regel		3200–3700	Bulbs	S-Allyl-L-cysteine sulfoxide	Antioxidant, Cardiovascular health benefits	[234]
4.	*Pistacia integerrima* L.	Anacardiaceae	800–2200	Fruits	Gallic acid, Quercetin	Antioxidant, Anti-inflammatory	[235]
5.	*Angelica glauca* Edgew.	Apiaceae	2000–3800	Roots	Angelicin, Umbelliferone	Antioxidant, Hepatoprotective	[236]
6.	*Bupleurum falcatum* L		2130–3500	Roots	Saikosaponins	Anti-inflammatory, Hepatoprotective	[237]
7.	*Chaerophyllum aromaticum* L.		2800–3200	Roots	Coumarin, Umbelliferone	Antioxidant, Anti-inflammatory	[238]
8.	*Ferula jaeschkeana* Vatke		2600–3000	Rhizomes	Ferutinin, Ferulenol	Antioxidant	[239]
9.	*Heracleum candicans* L.		1800–4000	Leaves, Stem Roots	Bergapten, Psoralen	Antioxidant, Anti-inflammatory	[240]
10.	*Pleurospermum brunonis* Benth. ex C.B Clarke		3000–4000	Leaves	Psoralen, Isopsoralen	Antioxidant, Anti-inflammatory	[241]
11.	*Selinum vaginatum* C.B. Clarke		2700–3800	Roots Bhutkeshi	Selinidin, Selinidiol	Antioxidant, Anti-inflammatory	[242]
12.	*Arisaema flavum* (Forsk.) Schott.	Araceae	2000–3400	Rhizome	Arisarumol	Antioxidant, Anti-inflammatory	[243]
13.	*Hedera nepalensis* C. Koch	Araliaceae	1500–3000	Leaves, Stems	Hederacoside C, Hederagenin	Antioxidant, Anti-inflammatory	[244]
14.	*Achillea millefolium* L.	Asteraceae	3200–3700	Leaves, Flowers	Apigenin, Luteolin	Antioxidant, Anti-inflammatory	[245]
15.	*Artemisia absinthium* L.		2000–3660	Whole plant	Absinthin, Anabsinthin	Antioxidant	[246]
16.	*Artemisia macrocephala* Jacq. ex Bess		3400–5500	Aerial parts	Artemisinin, Dihydroartemisinin	Antioxidant, Anticancer	[247]
17.	*Carduus nutans* L.		2600–3000	Leaves, Roots	Silymarin	Hepatoprotective, Antioxidant	[248]
18.	*Cichorium intybus* L.		2600–3000	Leaves, Roots	Inulin, Lactucin	Hepatoprotective, Hypoglycemic	[249]
19.	*Erigeron acris* L.		2600–3400	Roots	Quercetin, Kaempferol	Anti-inflammatory, Antioxidant	[250]
20.	*Inula cappa* DC.		2600–3500	Roots	Alantolactone, Isoalantolactone	Antioxidant, Anti-inflammatory	[251]
21.	*Inula racemosa* Hook. f.		2000–3100	Roots	Alantolactone, Isoalantolactone	Antioxidant, Anti-inflammatory	[252]
22.	*Jurinea dolomiaea* Boiss.		3000–4000	Roots	Jurineol, Jurineol acetate	Antioxidant, Anti-inflammatory	[253]
23.	*Jurinea macrocephala* DC.		3000–4000	Roots Leaves	Jurineol, Jurineol acetate	Antioxidant, Anti-inflammatory	[254]
24.	*Saussurea albescens* Hook. f. et. Thomson		2000–3600	Leaves	Costunolide, Eupatilin	Antioxidant, Anti-inflammatory	[255]
25.	*Saussurea costus* (Falc.) Lipsch.		2600–4000	Roots	Costunolide, Dehydrocostus lactone	Antioxidant, Anti-inflammatory	[256]
26.	*Saussurea gossypiphora* D. Don		4500–5300	Flowers	Saussureamine	Antioxidant, Anti-inflammatory	[257]
27.	*Scorzonera virgata* DC.		2700–4200	Leaves	Inulin, Scorzodioside B	Hepatoprotective, Hypoglycemic	[258]
28.	*Waldhemia glabra* (Decne.) Regel.		4000–5000	Aerial parts	Waldhemiol, Waldhemidin	Antioxidant, Anti-inflammatory	[259]
29.	*Waldhemia tomentosa* (Decne.) Regel.		3800–4500	Whole plant	Waldhemiol, Waldhemidin	Antioxidant, Anti-inflammatory	[260]
30.	*Impatiens sulcata* Wall.	Balsaminaceae	2000–3900	Whole plant	Lawsone	Antioxidant, Anti-inflammatory	[261]
31.	*Berberis lycium* Royle	Berberidaceae	1200–3000	Roots, stems	Berberine, Palmatine	Antioxidant, Antidiabetic	[262]
32.	*Betula utilis* D. Don	Betulaceae	2900–4000	Bark	Betulin, Betulinic acid	Antioxidant, Anti-inflammatory	[263]
33.	*Biebersteinia odora* Steph. ex Fish	Biebersteiniaceae	4200–5030	Rootstocks	Coumarin, Umbelliferone	Antioxidant, Anti-inflammatory	[264]
34.	*Arnebia benthamii* (Wall. ex G. Don.) Johnston	Boraginaceae	3000–3900	Roots	Alkannin, Shikonin	Antioxidant, Anti-inflammatory	[265]
35.	*Cynoglossum wallichii* G. Don		2600–3700	Leaves	Shikonin, Deoxyshikonin	Antioxidant, Anti-inflammatory	[266]
36.	*Cynoglossum zeylanicum* Thunb. ex Lehm. Brand.		2600–3350	Roots	Shikonin, Deoxyshikonin	Antioxidant, Anti-inflammatory	[266]
37.	*Myosotis silvatica* Ehrh. ex Hoffm.		3200–4200	Whole plant	Tannins, Flavonoids	Antioxidant, Anti-inflammatory	[267]
38.	*Onosma hispida* Wall. ex G. Don		2000–3400	Roots, Leaves	Alkannin, Shikonin	Antioxidant, Anti-inflammatory	[268]
39.	*Arabidopsis mollissma* (C. May.) N. Busch	Brassicaceae	3800–4300	Leaves	Sinapine, Sinapic acid	Antioxidant, Anti-inflammatory	[269]
40.	*Arabis nova* Vill.		3500–3900	Fruits	Glucosinolates	Antioxidant, Anticancer	[270]
41.	*Brassica rapa* L. ssp.		3200–4500	Whole plant	Glucosinolates	Antioxidant, Anticancer	[271]
42.	*Descurainia sophia* (L.) Webb. ex Prantl		2600–3500	Whole plant	Linalool, Thymoquinone	Antioxidant, Anti-inflammatory	[272]
43.	*Lepidium latifolium* L.		2500–4300	Aerial parts	Glucosinolates	Antioxidant	[273]
44.	*Nasturtium officinale* W.T. Ait. Hort.		2600–3500	Whole plant	Glucosinolates	Antioxidant	[274]
45.	*Sisymbrium orientale* L.		2600–3600	Seeds	Glucosinolates	Antioxidant	[275]
46.	*Sarcococca saligna* (D. Don) Muell.-Arg.	Buxaceae	1500–2300	Leaves, Stem	Sarcococcin	Antioxidant, Anti-inflammatory	[276]
47.	*Codonopsis clematidea* (Schrenk) C.B. Clarke	Campanulaceae	3000–3800	Flowers	Codonopsin, Codonopsidic acid	Antioxidant, Immunomodulatory	[277]
48.	*Codonopsis ovata* Benth.		2700–3200	Whole plant	Codonopsin, Codonopsidic acid	Antioxidant, Immunomodulatory	[278]
49.	*Cyananthus lobatus* Wall. ex Benth		3000–4000	Leaves, flowers	Cyanolobatolide	Antioxidant, Anti-inflammatory	[279]
50.	*Capparis himalayensis* Jafri	Capparaceae	2800–3300	Leaves	Flavonoids, Glucosinolates	Antioxidant	[280]
51.	*Lonicera hypoleuca* Decne.	Caprifoliaceae	2900–3100	Stem	Chlorogenic acid, Luteolin	Antioxidant, Anti-inflammatory	[281]
52.	*Lonicera quinquelocularis* Hardw.		2600–3500	Stems, Leaves, Fruit	Chlorogenic acid, Luteolin	Antioxidant, Anti-inflammatory	[282]
53.	*Viburnum cotinifolium* D. Don		2300–2600	Fruits	Iridoids, Flavonoids	Antioxidant, Anti-inflammatory	[283]
54.	*Viburnum grandiflorum* Buch-Ham. ex D. Don		2800–4300	Fruits, seeds	Iridoids, Flavonoids	Antioxidant, Anti-inflammatory	[283]
55.	*Cerastium cerastoides* (L.) Britt.	Caryophyllaceae	2000–4000	Whole plant	Tannins, Flavonoids	Antioxidant, Anti-inflammatory	[86]
56.	*Myosoton aquaticum* (L.) Moench		2000–2800	Leaves, Stem	Tannins, Flavonoids	Antioxidant, Anti-inflammatory	[284]
57.	*Silene vulgaris* (Moench) Garcke		2740–3450	Leaves, Twigs	Tannins, Flavonoids	Antioxidant, Anti-inflammatory	[285]
58.	*Stellaria media* (L.) Vill.		2600–3000	Leaves	Tannins, Flavonoids	Antioxidant, Anti-inflammatory	[286]
59.	*Chenopodium album* L.	Chenopodiaceae	350–4300	Leaves, Seeds	Saponins, Flavonoids	Antioxidant, Anti-inflammatory	[287]
60.	*Chenopodium foliosum* Wall.		2000–4000	Fruits	Saponins, Flavonoids	Antioxidant, Anti-inflammatory	[288]
61.	*Convolvulus arvensis* L.	Convolvulaceae	3000–4000	Flower buds	Alkaloids, Flavonoids	Antioxidant, Neuroprotective	[289]
62.	*Corylus jacquemontii* Decne.	Corylaceae	2000–3300	Seeds	Catechins, Quercetin	Antioxidant, Anti-inflammatory	[290]
63.	*Rosularia alpestris* (Kar. and Kir.) Boriss.	Crassulaceae	3000–4300	Whole plant	Phenolic compounds, Flavonoids	Antioxidant, Anti-inflammatory	[102]
64.	*Juniperus communis* L.	Cupressaceae	3000–4200	Needles	Monoterpenes, Flavonoids	Antioxidant	[291]
65.	*Juniperus indica* Bertol.		3500–4500	Wood	Monoterpenes, Flavonoids	Antioxidant	[292]
66.	*Cuscuta reflexa* Roxb.	Cuscutaceae	800–2500	Whole plant	Flavonoids, Alkaloids	Antioxidant, Hepatoprotective	[293]
67.	*Datisca cannabina* L.	Datiscaceae	2800–3200	Leaves, Roots	Tannins, Flavonoids	Antioxidant, Anti-inflammatory	[294]
68.	*Dioscorea deltoidea* Wall. ex Kunth	Dioscoreaceae	2000–2800	Tuber	Diosgenin, Dioscin	Antioxidant, Anti-inflammatory	[15]
69.	*Elaeagnus conferta* Roxb.	Elaeagnaceae	1500–2200	Fruits	Triterpenoids, Flavonoids	Antioxidant, Anti-inflammatory	[295]
70.	*Hippophae rhamnoides* L.		2600–3500	Fruits, Stem	Flavonoids, Vitamin C	Antioxidant, Immunomodulatory	[296]
71.	*Hippophae salicifolia* D. Don		2800–3500	Fruits	Flavonoids, Vitamin C	Antioxidant, Immunomodulatory	[297]
72.	*Cassiope fastigiata* (Wall.) D. Don	Ericaceae	3800–4600	Leaves	Polyphenols, Flavonoids	Antioxidant, Anti-inflammatory	[298]
73.	*Rhododendron anthopogon* D. Don		3200–4500	Leaves, Flowers	Rhododendrin, Ursolic acid	Antioxidant, Anti-inflammatory	[299]
74.	*Rhododendron arboretum* Sm.		2000–4000	Leaves, Flowers	Arbutin, Quercetin	Antioxidant, Anti-inflammatory	[300]
75.	*Rhododendron campanulatum* D. Don		3000–4300	Leaves	Arbutin, Quercetin	Antioxidant, Anti-inflammatory	[301]
76.	*Gentiana kurroo* Royle	Gentianaceae	1800–4200	Roots	Gentisin, Swertiamarin	Antioxidant, Hepatoprotective	[302]
77.	*Gentiana leucomelaena* Maxim. ex Kusn.		2500–5000	Whole plant	Gentisin, Swertiamarin	Antioxidant, Hepatoprotective	[303]
78.	*Gentiana moorcroftiana*		2700–5000	Leaves	Gentisin, Swertiamarin	Antioxidant, Hepatoprotective	[304]
79.	*Gentiana tianshanica* Rupr.		3900–3900	Whole plant	Gentisin, Swertiamarin	Antioxidant, Hepatoprotective	[305]
80.	*Gentiana tubiflora* (G. Don) Grirseb.		4000–5300	Whole plant	Gentisin, Swertiamarin	Antioxidant, Hepatoprotective	[306]
81.	*Gentianopsis detonsa* (Rottb.) Ma		2700–4200	Whole plant	Gentisin, Swertiamarin	Antioxidant, Hepatoprotective	[303]
82.	*Gentianopsis paludosa* (Hook.) Ma		3000–4000	Whole plant	Gentisin, Swertiamarin	Antioxidant, Hepatoprotective	[307]
83.	*Swertia chirayita* (Roxb. ex Fleming) Karst.		1500–3000	Whole plant	Amarogentin, Swertiamarin	Antioxidant, Hepatoprotective	[308]
84.	*Geranium pratense* L.	Geraniaceae	2680–3900	Whole plant	Geraniin, Tannins	Antioxidant, Anti-inflammatory	[309]
85.	*Geranium wallichianum* D. Don ex Sweet		2600–3980	Whole plant	Geraniin, Tannins	Antioxidant, Anti-inflammatory	[310]
86.	*Juglans regia* L.	Juglandaceae	1000–3300	Leaves, seeds	Juglone, Quercetin	Antioxidant, Anti-inflammatory	[311]
87.	*Lamium album* L.	Lamiaceae	1500–2400	Roots, Rhizomes	Rosmarinic acid, Flavonoids	Antioxidant, Anti-inflammatory	[312]
88.	*Origanum vulgare* L		1800–3600	Leaves, Stems	Carvacrol, Thymol	Antioxidant	[313]
89.	*Phlomis bracteosa* Royle ex Benth.		3200–4400	Whole plant	Ursolic acid	Antioxidant, Anti-inflammatory	[314]
90.	*Salvia nubicola* Wall. ex Sweet		2000–2700	Roots, Leaves	Salvianolic acid, Rosmarinic acid	Antioxidant, Anti-inflammatory	[315]
91.	*Astragalus bicuspis* Fischer	Leguminosae	3100–3500	Whole plant	Astragaloside IV	Antioxidant, Immunomodulatory	[316]
92.	*Astragalus candolleanus* Royle		3000–4000	Roots	Astragaloside IV	Antioxidant, Immunomodulatory	[317]
93.	*Astragalus grahamianus* Royle ex Benth.		3000–3500	Whole plant	Astragaloside IV	Antioxidant, Immunomodulatory	[318]
94.	*Astragalus himalayanus* Klotzsch		3200–4400	Flowers Seeds	Astragaloside IV	Antioxidant, Immunomodulatory	[319]
95.	*Astragalus strobiliferus* Royle		3000–4000	Roots	Astragaloside IV	Antioxidant, Immunomodulatory	[320]
96.	*Astragalus zanskarensis* Benth. ex Bunge		3200–4600	Roots	Astragaloside IV	Antioxidant, Immunomodulatory	[321]
97.	*Cicer microphyllum* Benth.		3200–4600	Aerial parts,	Flavonoids, Saponins	Antioxidant, Anti-inflammatory	[322]
98.	*Desmodium elegans* DC.		2000–4000	Leaves	Flavonoids, Alkaloids	Anti-inflammatory	[323]
99.	*Lotus corniculatus* L.		2500–3400	Whole plant	Rutin, Quercetin	Antioxidant, Anti-inflammatory	[324]
100.	*Medicago falcata* L.		2700–3500	Aerial parts	Isoflavones, Saponins	Antioxidant, Anti-inflammatory	[325]
101.	*Trifolium pratense* L.		2600–3800	Whole plant	Formononetin, Biochanin A	Antioxidant	[326]
102.	*Trifolium repens* L.		2600–3200	Whole plant	Trifoside, Genistein	Antioxidant, Anti-inflammatory	[327]
103.	*Trigonella emodi* Benth.		2600–3800	Whole plant	Trigonelline, Diosgenin	Antioxidant, Antidiabetic, Hypolipidemic	[328]
104.	*Vicia sativa* L.		2600–3000	Whole plant	Vicine, Convicine	Antioxidant, Antidiabetic	[329]
105.	*Eremurus himalaicus* Baker	Liliaceae	3200–4500	Fruits	Steroidal saponins	Anti-inflammatory, Immunomodulatory	[330]
106.	*Viscum album* L.	Loranthaceae	2000–3000	Bark	Viscotoxins, Lectins	Antioxidant, Immunomodulatory	[331]
107.	*Malva neglecta* Wallr.	Malvaceae	2600–4500	Whole plant	Mucilage	Antioxidant, Anti-inflammatory	[332]
108.	*Malva verticillata* L.		2500–3800	Seeds	Mucilage	Antioxidant, Anti-inflammatory	[333]
109.	*Morus serrata* Roxb.	Moraceae	2000–2300	Leaves, Fruits	Morin, Resveratrol	Antioxidant, Anti-inflammatory	[334]
110.	*Morina coulteriana* Royle	Morinaceae	3000–3700	Flowers	Morin	Antioxidant, Anti-inflammatory	[335]
111.	*Morina longifolia* Wall. ex DC.		3000–4300	Roots, Flowers	Morin	Antioxidant, Anti-inflammatory	[336]
112.	*Jasminum officinale* L.	Oleaceae	1800–4000	Leaves Stems	Jasmonic acid, Quercetin	Antioxidant, Anti-inflammatory	[337]
113.	*Epilobium angustifolium* L.	Onagraceae	3000–4700	Roots	Oenothein B, Quercetin	Antioxidant, Anti-inflammatory	[338]
114.	*Oenothera glazioviana* Micheli		2000–2700	Whole plant	Linoleic acid, Gamma-linolenic acid	Antioxidant, Anti-inflammatory	[339]
115.	*Dactylorhiza hatagirea* D. Don	Orchidaceae	3000–3800	Rhizome	Phenanthrenes	Antioxidant, Anti-inflammatory	[340]
116.	*Meconopsis aculeata* Royle	Papaveraceae	2400–4200	Whole plant	Alkaloids, Flavonoids	Antioxidant, Anti-inflammatory	[341]
117.	*Parnassia nubicola* Hook. f.	Parnassiaceae	1900–3400	Roots	Parnassiol	Antioxidant, Hepatoprotective, Anti-inflammatory	[342]
118.	*Cedrus deodara* (Royle ex D. Don)	Pinaceae	1600–3000	Wood	Deodarone, Cedrol	Antioxidant	[343]
119.	*Pinus gerardiana* Wall. ex Lambert.		2500–3000	Fruits/Kernels	Pinene, Pinenes	Antioxidant, Anti-inflammatory	[344]
120.	*Pinus nigra* Aiton, Hort. Kew. [W. Aiton]		1300–2200	Fruits/Kernels	Pinene, limonene borneol	Antioxidant, Anti-inflammatory	[345]
121.	*Plantago depressa* Willd.	Plantaginaceae	2000–4500	Whole plant	Glycosides, Flavonoids	Antioxidant, Anti-inflammatory	[346]
122.	*Plantago major* L.		2000–2800	Leaves, Roots,	Aucubin, Ursolic acid	Antioxidant, Anti-inflammatory	[347]
123.	*Bistorta vaccinifolia* (Wall. ex Meisn.) Greene	Polygonaceae	3000–4600	Whole plant	Tannins, Flavonoids	Antioxidant, Anti-inflammatory	[348]
124.	*Koenigia delicatula* (Meisn.) H. Hara		3000–4500	Stems	Tannins, Flavonoids	Antioxidant, Anti-inflammatory	[349]
125.	*Oxyria digyna* Hill		2600–5300	Whole plant	Oxycoumarins	Antioxidant, Anti-inflammatory	[350]
126.	*Polygonum alpinum* Allioni.		1500–2400	Stems, Leaves	Rutin, Quercetin	Antioxidant, Anti-inflammatory	[351]
127.	*Polygonum aviculare* L.		2000–4200	Flower buds	Polyphenols, Flavonoids	Antioxidant, Anti-inflammatory	[352]
128.	*Polygonum plebejum* R.Br.		1000–4000	Whole plant	Polyphenols, Flavonoids	Antioxidant, Anti-inflammatory	[353]
129.	*Polygonum pubescens* Blume		1500–3700	Roots	Polyphenols, Flavonoids	Antioxidant, Anti-inflammatory	[354]
130.	*Polygonum tortuosum* D. Don		3600–4900	Young peduncle	Polyphenols, Flavonoids	Antioxidant, Anti-inflammatory	[352]
131.	*Rheum australe* D. Don		3300–5200	Roots	Anthraquinones, Tannins	Antioxidant	[355]
132.	*Rheum spiciforme* Royle		4000–5000	Peduncle	Anthraquinones, Tannins	Antioxidant	[356]
133.	*Rumex acetosa* L.		1500–4000	Leaves	Anthraquinones, Tannins	Antioxidant	[357]
134.	*Rumex hestatus* D. Don		1500–3700	Leaves, Stem	Anthraquinones, Tannins	Antioxidant	[358]
135.	*Rumex nepalensis* Spreng.		1200–4000	Roots	Anthraquinones, Tannins	Antioxidant	[359]
136.	*Aconitum heterophyllum* Wall. ex Royle	Ranunculaceae	3200–4500	Roots	Aconitine, Pseudoaconitine	Antioxidant, Anti-inflammatory	[360]
137.	*Aconitum rotundifolium* Kar. and Kir.		3500–4800	Stem	Aconitine, Pseudoaconitine	Antioxidant, Anti-inflammatory	[361]
138.	*Aconitum violaceum* Jacq. ex Stapf		3200–4400	Roots	Aconitine, Pseudoaconitine	Antioxidant, Anti-inflammatory	[362]
139.	*Aconitum heterophyllum* Wall. ex Royle.		2000–4000	Roots	Aconitine, atisine, heteratisine, hetisine	Antioxidant, Anti-inflammatory	[363]
140.	*Anemone rivularis* Buch. Ham. ex DC.		2400–3300	Leaves, Roots	Saponins, Tannins	Antioxidant, Anti-inflammatory	[364]
141.	*Aquilegia fragrans* Benth.		2900–3500	Whole plant	Alkaloids, Flavonoids	Antioxidant, Anti-inflammatory	[365]
142.	*Aquilegia moorcroftiana* Wall. ex Royle		3300–3700	Twigs	Alkaloids, Flavonoids	Antioxidant, Anti-inflammatory	[366]
143.	*Caltha palustris* L.		3020–3500	Leaves, Roots	Protoanemonin	Antioxidant, Anti-inflammatory	[367]
144.	*Clematis grata* Wall.		2000–2600	Leaves	Clematichinenoside	Antioxidant, Anti-inflammatory	[368]
145.	*Clematis ladakhiana* C. Grey-Wilson		3200–3900	Roots Shoots	Clematichinenoside	Antioxidant, Anti-inflammatory	[369]
146.	*Clematis orientalis* L.		3400–5200	Whole plant	Clematichinenoside	Antioxidant, Anti-inflammatory	[370]
147.	*Crataegus songarica* K. Koch		1500–2000	Fruits, Leaves	Flavonoids, Triterpenes	Antioxidant, Cardioprotective	[371]
148.	*Fragaria nubicola* Lindl.		2500–3900	Fruit, Roots	Anthocyanins, Ellagic acid	Antioxidant	[372]
149.	*Geum elatum* Wall. ex G. Don		3500–4500	Roots	Tannins, Flavonoids	Antioxidant, Anti-inflammatory	[373]
150.	*Potentilla atrisanguinea* Lodd. var. *argyrophylla* (Wall. ex Lehm.) Griers. and Long		3000–4500	Roots	Tannins, Flavonoids	Antioxidant, Anti-inflammatory	[374]
151.	*Potentilla eriocarpa* Wall. ex Lehm.		3000–5000	Whole plant	Tannins, Flavonoids	Antioxidant, Anti-inflammatory	[375]
152.	*Potentilla fulgens* Wall.		2000–3200	Roots	Tannins, Flavonoids	Antioxidant, Anti-inflammatory	[376]
153.	*Potentilla nubicola* Lindl. ex Lacaita		2900–4000	Fruits	Tannins, Flavonoids	Antioxidant, Anti-inflammatory	[377]
154.	*Prinsepia utilis* Royle		1800–3000	Seeds, Roots	Triterpenes, Flavonoids	Antioxidant, Hepatoprotectiv	[378]
155.	*Pyracantha crenulata* (D. Don) Roemer		1000–2600	Fruits	Flavonoids, Triterpenes	Antioxidant, Anti-inflammatory	[379]
156.	*Pyrus lanata* D. Don.		2700–3400	Fruits	Triterpenes, Flavonoids	Antioxidant, Hepatoprotective	[380]
157.	*Rosa brunonii* Lindl.		2100–4500	Flowers	Anthocyanins, Flavonoids	Antioxidant, Anti-inflammatory	[381]
158.	*Rosa webbiana* Wall. ex Royle		3000–3800	Fruits, Stem, Flowers	Anthocyanins, Flavonoids	Antioxidant, Anti-inflammatory	[382]
159.	*Rubus ellipticus* Sm.		1800–2600	Fruits	Anthocyanins, Ellagic acid	Antioxidant	[383]
160.	*Rubus niveus* Thunb.		2000–2800	Fruits	Anthocyanins, Ellagic acid	Antioxidant	[384]
161.	*Spiraea canescens* D. Don		2600–4000	Stem	Tannins, Flavonoids	Antioxidant, Anti-inflammatory	[385]
162.	*Rubia cordifolia* L.	Rubiaceae	1800–3000	Leaves, Stem, Roots	Anthraquinones, Tannins	Antioxidant, Anti-inflammatory	[386]
163.	*Euphrasia flabellate* Pennell	Scrophulariaceae	3000–4000	Whole plant	Iridoid glycosides, Flavonoids	Antioxidant, Anti-inflammatory	[387]
164.	*Euphrasia paucifolia* Wettst.		3000–4300	Leaves	Iridoid glycosides, Flavonoids	Antioxidant, Anti-inflammatory	[388]
165.	*Picrorhiza kurroa* Royle ex Benth.		3000–4000	Roots	Picroside I, Picroside II	Antioxidant, Hepatoprotective	[389]
166.	*Scrophularia calycina* Benth.		3000–4000	Whole plant	Iridoid glycosides, Flavonoids	Antioxidant, Anti-inflammatory	[390]
167.	*Scrophularia decomposita* Royle ex Benth.		3000–4200	Leaves	Iridoid glycosides, Flavonoids	Antioxidant, Anti-inflammatory	[391]
168.	*Urtica dioica* Jacq. ex Wedd.	Urticaceae	2000–3000	Leaves	Acetylcholine, Histamine	Anti-inflammatory	[392]

## 5. Challenges of Using High-Altitude Phytochemicals in Medicine

### 5.1. Challenges in Extraction and Utilization

Extracting and utilizing compounds from high-altitude medicinal plants hold immense promise for various practical applications, yet it also presents formidable challenges and limitations that demand thoughtful consideration and innovative solutions [393]. These plants, thriving in extreme environments that are characterized by harsh climatic conditions and often rare or endemic species, offer unique bioactive compounds that could revolutionize pharmaceutical, nutraceutical, and cosmetic industries. However, factors such as environmental harshness, slow growth rates, seasonal variability in compound production, and the complexity of chemical profiles pose significant hurdles. The limiting factors when utilizing high-altitude-plant-derived compounds in modern health care are discussed as follows [394].

#### 5.1.1. Harsh Environmental Conditions

High-altitude environments are characterized by extreme temperatures, strong UV radiation, and variable weather conditions. Accessing and cultivating these plants can be challenging due to these harsh conditions, which can affect plant growth, availability, and quality of the compounds extracted [395].

#### 5.1.2. Low Biomass and Slow Growth

Many high-altitude plants have slow growth rates and produce low biomass. This makes it difficult to obtain sufficient quantities of the desired compounds for large-scale extraction and commercial applications [396].

#### 5.1.3. Species Rarity and Endemism

Some high-altitude plants are rare, endemic to specific regions, or protected due to conservation concerns. Harvesting these plants for medicinal compounds may raise ethical and sustainability issues if not managed carefully [397].

#### 5.1.4. Seasonal Variability

The growth and production of bioactive compounds in high-altitude plants can be highly seasonal and dependent on weather patterns. This variability makes it challenging to maintain a consistent quality and quantity of extracts throughout the year [398].

#### 5.1.5. Complex Chemistry

High-altitude plants often contain complex chemical profiles with multiple bioactive compounds. Identifying, isolating, and characterizing the specific compounds responsible for medicinal properties can be difficult and require sophisticated analytical techniques [395].

#### 5.1.6. Extraction Efficiency

Extracting bioactive compounds from high-altitude plants can be inefficient due to factors such as low compound concentrations, the presence of interfering substances, and extraction methods that may not be optimized for these specific plant materials [395].

#### 5.1.7. Cultural and Traditional Knowledge

Utilizing medicinal plants from high-altitude regions often requires understanding traditional knowledge and practices. Integrating modern extraction techniques with traditional wisdom can be challenging but is essential for sustainable use [399].

Addressing these challenges requires interdisciplinary approaches that blend botanical expertise, ecological understanding, advanced extraction technologies, and respect for local traditions and environmental sustainability. Research and development efforts aimed at overcoming these limitations are crucial for harnessing the potential of high-altitude medicinal plants for practical applications in healthcare and beyond.

### 5.2. Regulatory Challenges

Regulatory challenges encompass navigating diverse international regulations governing the use of natural products in healthcare, which vary widely in terms of safety standards, labelling requirements, and permissible claims. Compliance with these regulations is crucial to establish the credibility and legality of medicinal products derived from high-altitude plants. Moreover, rigorous quality control measures are indispensable for guaranteeing the batch-to-batch consistency, purity, and potency of phytochemical antioxidants. Variations in environmental factors, such as altitude, climate, and soil composition, can significantly influence the composition and concentration of bioactive compounds in these plants, underscoring the need for robust analytical methods, standardized protocols and regulatory bodies, stringent quality control, rigorous clinical trials, protected intellectual property, and traditional knowledge that is integrated with scientific advancements [400].

To establish robust safety assessment plans and regulatory frameworks for manufacturing herbal medicines, various global regulatory bodies have issued guidance documents. These include the International Life Sciences Institute, Washington, DC, USA; the International Union of Pure and Applied Chemistry, North Carolina, USA; the European Medicines Agency, Amsterdam, The Netherlands and the European Food Safety Authority in Parma, Italy [401]. In the USA, the sale and purchase of herbal medicines falls under the regulation of the Dietary Supplement Health and Education Act of 1994 [402]. In the European Union, national regulatory bodies like the Committee on Herbal Medicinal Products (HMPC), part of the European Medicines Agency, oversee the production and marketing of herbal drugs. Similarly, Canada operates under the Natural Health Products Regulations (NHPR), and Australia relies on the Therapeutic Goods Administration (TGA) to regulate the manufacturing and marketing of herbal drugs [403,404]. In India, the Ministry of AYUSH serves as the regulatory authority responsible for issuing licenses for the manufacturing and marketing of herbal drugs [405].

By addressing these regulatory and quality control challenges proactively, researchers and practitioners can ensure that phytochemical antioxidants from high-altitude plants meet the stringent safety and efficacy standards required for medical use. This approach not only enhances the credibility of natural products but also fosters trust among healthcare providers and patients, facilitating their broader adoption and contribution to improving health outcomes. Therefore, future studies should prioritize these critical considerations in order to accelerate the translation of research findings into impactful medical applications.

## 6. Future Prospects

### 6.1. Dietary Phytochemicals as Antioxidants

Dietary phytochemicals, encompassing a diverse array of compounds, like flavonoids, polyphenols, and carotenoids, play a pivotal role as antioxidants in human health [406]. These compounds are specifically derived from plant-based foods consumed as part of a diet, distinguishing them from phytochemicals in general, which include those found in plants but may not necessarily be consumed through dietary sources. By scavenging free radicals, chelating metal ions, and activating endogenous antioxidant enzymes, dietary phytochemicals mitigate oxidative stress and prevent the cellular damage associated with chronic diseases such as cancer [407], cardiovascular diseases [2], neurodegenerative disorders [2], gastrointestinal disorders [2], metabolic disorders and ageing [408]. The following examples highlight the antioxidant prowess of dietary phytochemicals like curcumin, resveratrol, quercetin, and epigallocatechin gallate. Curcumin, abundant in turmeric, not only scavenges free radicals but also exhibits potent anti-inflammatory effects, making it a promising candidate for conditions like Alzheimer’s disease and cancer [409]. Resveratrol, found in red grapes and wine, not only scavenges free radicals but also improves endothelial function and reduces oxidative damage in cardiovascular tissues, potentially reducing the risk of heart disease [410]. Quercetin, present in various fruits and vegetables, not only scavenges free radicals but also modulates signalling pathways involved in oxidative stress and inflammation, offering protection against chronic inflammatory diseases [180]. EGCG, abundant in green tea, not only scavenges free radicals but also induces antioxidant enzymes and inhibits oxidative damage to DNA and proteins, contributing to cancer prevention and overall longevity [411]. These compounds exhibit multifaceted antioxidant mechanisms, thereby contributing to overall health and longevity. 

Incorporating a variety of dietary phytochemical-rich plant foods into the diet offers a promising strategy for disease prevention and promoting optimal wellbeing. Ongoing research into novel dietary phytochemicals continues to expand our understanding of their antioxidant properties, fuelling the development of dietary interventions for combating oxidative stress-related disorders. Biotechnological advances hold the promise of enhancing production and bioavailability, while personalized nutrition approaches will tailor antioxidant interventions to individual needs. Overall, the future of plant-based antioxidants is promising, offering innovative solutions for improving health and wellbeing on a global scale.

### 6.2. Novel Delivery Systems for Sustained Release

Innovative delivery systems for the sustained release of phytochemical antioxidants are poised to revolutionize the field of nutraceuticals and pharmaceuticals. These systems, ranging from nanoparticles to encapsulation techniques, offer enhanced stability, bioavailability, and targeted delivery of antioxidant compounds [412]. Nanoparticle-based carriers, such as liposomes, polymeric nanoparticles, and solid lipid nanoparticles, enable the controlled release and protection of phytochemicals during digestion, ensuring optimal absorption and efficacy. Encapsulation methods, including microencapsulation and nano-emulsions, provide a protective matrix for phytochemicals, preventing degradation and enabling sustained release in the gastrointestinal tract. These delivery systems not only improve the therapeutic potential of phytochemical antioxidants but also offer opportunities for functional food fortification and personalized supplementation strategies [413]. As research in this area continues to advance, novel delivery systems hold promise for addressing complex health challenges and optimizing the preventive and therapeutic effects of phytochemical antioxidants. 

One prominent innovative delivery system used for phytochemical antioxidants is nanoemulsions. Nanoemulsions are colloidal dispersions of oil and water stabilized by surfactants or emulsifiers, with droplet sizes typically in the nanometre range. These delivery systems offer several advantages for phytochemical encapsulation and controlled release, including enhanced solubility, stability, and bioavailability. By encapsulating phytochemicals within nanoemulsion droplets, their exposure to harsh environmental conditions, such as pH changes and enzymatic degradation in the gastrointestinal tract, can be minimized, leading to improved absorption and efficacy [414]. Nanoemulsions have been successfully employed to deliver various antioxidant compounds from plant sources, such as polyphenols and carotenoids, in functional foods, dietary supplements, and pharmaceutical formulations [415]. Their versatility and effectiveness make nanoemulsions a promising strategy for harnessing the potential of phytochemical antioxidants in combating oxidative stress-related diseases.

Nanoparticles represent another prominent and versatile delivery system for phytochemical antioxidants. Nanoparticles, including liposomes, polymeric nanoparticles, and solid lipid nanoparticles, offer numerous advantages, such as controlled release, enhanced stability, and the targeted delivery of antioxidants [416]. Liposomes encapsulate hydrophilic and hydrophobic phytochemicals within their aqueous core or lipid bilayer. Polymeric nanoparticles, on the other hand, provide a customizable platform for encapsulating phytochemicals through techniques like nanoprecipitation or emulsion solvent evaporation. Additionally, solid lipid nanoparticles offer improved stability and sustained release of antioxidants due to their lipid matrix [417]. These nanoparticle-based delivery systems enable the efficient protection of phytochemicals from degradation, prolonged circulation time, and enhanced cellular uptake, making them promising candidates for pharmaceutical and nutraceutical applications in combating oxidative stress and related diseases.

Innovative delivery systems represent a promising avenue for enhancing the efficacy and applicability of phytochemical antioxidants. Microencapsulation, for instance, offers a means by which to encapsulate antioxidants within protective matrices, safeguarding them from degradation while enabling controlled release. This approach ensures sustained antioxidant activity, addressing issues of stability and bioavailability [418]. Additionally, smart delivery systems, incorporating molecular sensors and precision targeting mechanisms, hold potential for the precise and efficient delivery of antioxidants to sites of oxidative stress. Nanoparticle-based carriers, such as liposomes and polymeric nanoparticles, provide further opportunities for enhanced delivery and cellular uptake of antioxidants. These systems offer controlled release kinetics and the ability to tailor delivery to specific tissues or cells, optimizing therapeutic outcomes. Furthermore, the development of nanogel-based delivery systems presents a promising approach for topical antioxidant delivery, offering improved skin penetration and localized antioxidant activity [419]. As research in this field progresses, these innovative delivery systems hold significant promise for advancing the therapeutic utility of phytochemical antioxidants in combating oxidative stress-related diseases.

## 7. Methodology

In this study, two databases, namely PubMed and DOAJ, were searched particularly by using the following specific keywords: “Oxidative stress and health disorders”, “High altitude medicinal plants” and “plant antioxidants.” Collectively, a total number of 30,924 articles, both reviews as well as original research articles, published from the year 1930 to 2023, were identified. The selection was meticulously undertaken as per the focus of the present study and only those articles published in peer-reviewed journals were included in the study to ensure the quality of the work.

Moreover, the chemical structures and formulae of phytocompounds were sourced from PubChem by using their common as well as IUPAC names (wherever necessary). The botanical names of the plants mentioned in the study have been cross verified with the International Plant Names Index (IPNI).

## 8. Conclusions

As a prominent contributing factor, oxidative stress poses serious health hazards, including cardiovascular diseases; cancers; neurological disorders, such as Parkinson’s and Alzheimer’s diseases; and metabolic disorders, which ultimately lead to mortality. This phenomenon arises from the generation of reactive oxygen and nitrogen species within the human body, causing damage to crucial biomolecules such as DNA, proteins, and lipids. Moreover, oxidative stress disrupts gene expression levels, resulting in chronic health conditions. The rise in oxidative stress levels can be attributed to environmental and lifestyle changes prevalent today. Factors like high pollution levels in the environment, along with unhealthy habits such as alcohol consumption, cigarette smoking, and poor dietary patterns, contribute significantly to oxidative stress and its associated health disorders. 

Given the detrimental effects of oxidative stress, its prevention and inhibition have become increasingly crucial. Antioxidants play a key role in combating oxidative stress and mitigating its detrimental effects. They can be produced naturally within the body or obtained through dietary or other supplements. Natural sources of antioxidants, particularly those derived from plants and their bioactive compounds, have emerged as a beneficial approach due to their immense versatility, bioavailability and minimal side effects. Phytochemicals, including polyphenols, flavonoids, ascorbic acid, tocopherols, and tocotrienols, possess inherent abilities to neutralize toxic oxygen species such as hydrogen peroxide and singlet oxygen radicals, thus counteracting oxidative stress. 

Plants produce a plethora of secondary metabolites, with a range of biological effects, including antioxidants. Further, plants thriving in high-altitude regions experience numerous environmental stresses that stimulate the production of these phytochemicals in substantial quantities. However, despite advances in research, much of the available biodiversity among the plants and their phytocompounds is still unexplored. This necessitates further exploration of the antioxidant properties of high-altitude medicinal plants to unlock their full therapeutic potential. By investing in interdisciplinary research initiatives, the wealth of natural antioxidants present in these plants could be harnessed with the development of innovative approaches in preventive healthcare and drug discovery. Collaboration between scientists, indigenous communities, and policymakers is essential to promote the sustainable utilization of plant resources and improve global health outcomes. It is imperative that we prioritize the exploration of high-altitude medicinal plants in order to address the growing burden of oxidative stress-related diseases and contribute to the preservation of biodiversity and traditional knowledge for the improvement of human health.
